# Nutritional Strategies for Selected MASLD Outcomes: Metabolic Remodeling, Liver-Aging Biomarkers, and Public Health Priorities

**DOI:** 10.3390/metabo16100795

**Published:** 2026-10-19

**Authors:** Hairong Cai, Huiling Cai, Tianliang Cai, Huiying Zhao, Qiyue Wang, Chang Liu, Wei Wu, Xingui Cai

**Affiliations:** 1 The Second Clinical College of Guangzhou University of Chinese Medicine, Guangzhou 510120, China; caihairong@gzucm.edu.cn (H.C.); 2388867762@gzucm.edu.cn (H.C.); a13654112671@163.com (T.C.); 2 School of Exercise and Health, Shanghai University of Sport, Shanghai 200438, China; zhaohy1237@163.com; 3 School of Competitive Sports, Beijing Sport University, Beijing 100084, China; 2024010238@bsu.edu.cn; 4 School of Sport Science, Beijing Sport University, Beijing 100084, China; c.liu@bsu.edu.cn; 5 School of Athletic Performance, Shanghai University of Sport, Shanghai 200438, China; wuwei1@sus.edu.cn

**Keywords:** metabolic dysfunction-associated steatotic liver disease, metabolomics, metabolic remodeling, biological aging, precision nutrition, epigenetic clocks, public health, health equity

## Abstract

Metabolic dysfunction-associated steatotic liver disease (MASLD) connects nutritional exposures with cardiometabolic risk and vulnerability during aging. Nutrition provides an actionable foundation for care, while metabolomics may clarify variation in dietary responses. This critical narrative review integrates nutritional trials, metabolic and aging studies, and clinical guidance from PubMed, Web of Science, Scopus, and Google Scholar through September 2026 using a documented narrative-search and selection process. Evidence is differentiated by population, original disease definition, design, tissue source, biomarker role, and outcome. Sustained weight reduction and selected dietary interventions, including Mediterranean-style and other high-quality dietary patterns, can improve selected hepatic or cardiometabolic outcomes, although comparative superiority is inconsistent. Lipid handling, nutrient sensing, mitochondrial function, and microbial metabolites provide mechanistic context. Metabolomic signatures characterize dietary exposure and metabolic adaptation, whereas direct evidence that nutritional treatment slows liver-specific biological aging remains absent: such evidence would require a defined MASLD population, a nutritional intervention, and a liver-tissue or independently validated liver-specific aging endpoint. Nutritional effects on aging measures are heterogeneous and largely derived from populations not selected for MASLD. The synthesis identifies public health priorities: reducing cumulative dietary risk, integrating nutritional care with fibrosis and cardiometabolic assessment, preserving muscle function, and expanding access to sustained support. A qualification framework distinguishes exposure, association, prognosis, response, and candidate surrogate roles while incorporating cost and equity. Accessible nutritional care can improve selected MASLD outcomes now; aging-informed precision nutrition remains investigational until molecular testing improves decisions and durable health benefits beyond established assessment.

## 1. Introduction

Metabolic dysfunction-associated steatotic liver disease (MASLD) places nutritional prevention at the intersection of liver disease, cardiometabolic health, and population aging. A global synthesis estimated an approximately 30% prevalence of nonalcoholic fatty liver disease (NAFLD), with substantial geographic and temporal variation [1]. The 2023 consensus defined MASLD by hepatic steatosis and at least one cardiometabolic risk factor, while retaining steatohepatitis as a distinct pathological process [2]. Historical NAFLD studies remain central to this review, although their eligibility criteria and prevalence estimates do not reproduce the MASLD definition exactly. The scale of this burden makes accessible, sustained nutritional care a public health priority.

The public health objective extends from reducing steatosis to preventing complications and preserving function. In a prospective study of 1773 adults, advanced fibrosis identified patients at greater risk of liver-related complications and death [3]. European guidance and American Association for the Study of Liver Diseases (AASLD) guidance link management to fibrosis assessment, cardiometabolic comorbidity, and sustained lifestyle change [4,5]. Mediterranean dietary trials also demonstrate cardiovascular benefits in high-risk populations, supporting dietary choices that address risks shared by many patients with MASLD [6,7]. These cardiovascular trials do not establish MASLD-specific event reduction, but they strengthen the rationale for integrated nutritional care.

Metabolic remodeling connects dietary exposures to differences in hepatic resilience. Nutrient-sensing dysregulation, mitochondrial dysfunction, cellular senescence, impaired macroautophagy, inflammation, and dysbiosis are shared features of aging biology and chronic nutritional stress [8]. Metabolomics can characterize substrates and signaling molecules within these pathways, while epigenetic and proteomic measures provide complementary information about biological state. Distinguishing exposure, adaptation, injury, and aging is essential because a diet-responsive metabolite is not automatically a marker of healthier aging.

A recent systematic review synthesized 19 observational studies relating biological aging to the MASLD spectrum [9]. The unresolved translational issue is how metabolic and aging measurements can improve nutritional decisions and outcomes. This review connects nutritional efficacy, metabolic mechanisms, and biomarker qualification to a common public health objective: effective care that remains accessible across the life course. It separates biomarker responsiveness, modification of an aging-related process, and surrogate-endpoint validity. The resulting framework identifies established dietary priorities, defines appropriate uses of molecular measurements, and specifies the evidence needed for equitable implementation.

### Distinct Aging Constructs Require Distinct Interpretation

Here, *liver biological aging* denotes age-related loss of hepatic resilience and function as a biological hypothesis, without assuming that a single validated measure captures it. Four measurement categories are kept separate throughout: liver-tissue aging measures obtained from hepatic samples; systemic aging measures associated with MASLD, including blood epigenetic clocks and DunedinPACE; liver-enriched circulating signatures, such as organ-enriched plasma protein models; and clinical or prognostic composite age indices built from routine measurements. The term “liver age” is reserved for a named liver-directed model and is qualified by its tissue source or construction. Association with MASLD alone does not make a systemic measure liver-specific.

**Figure 1:**
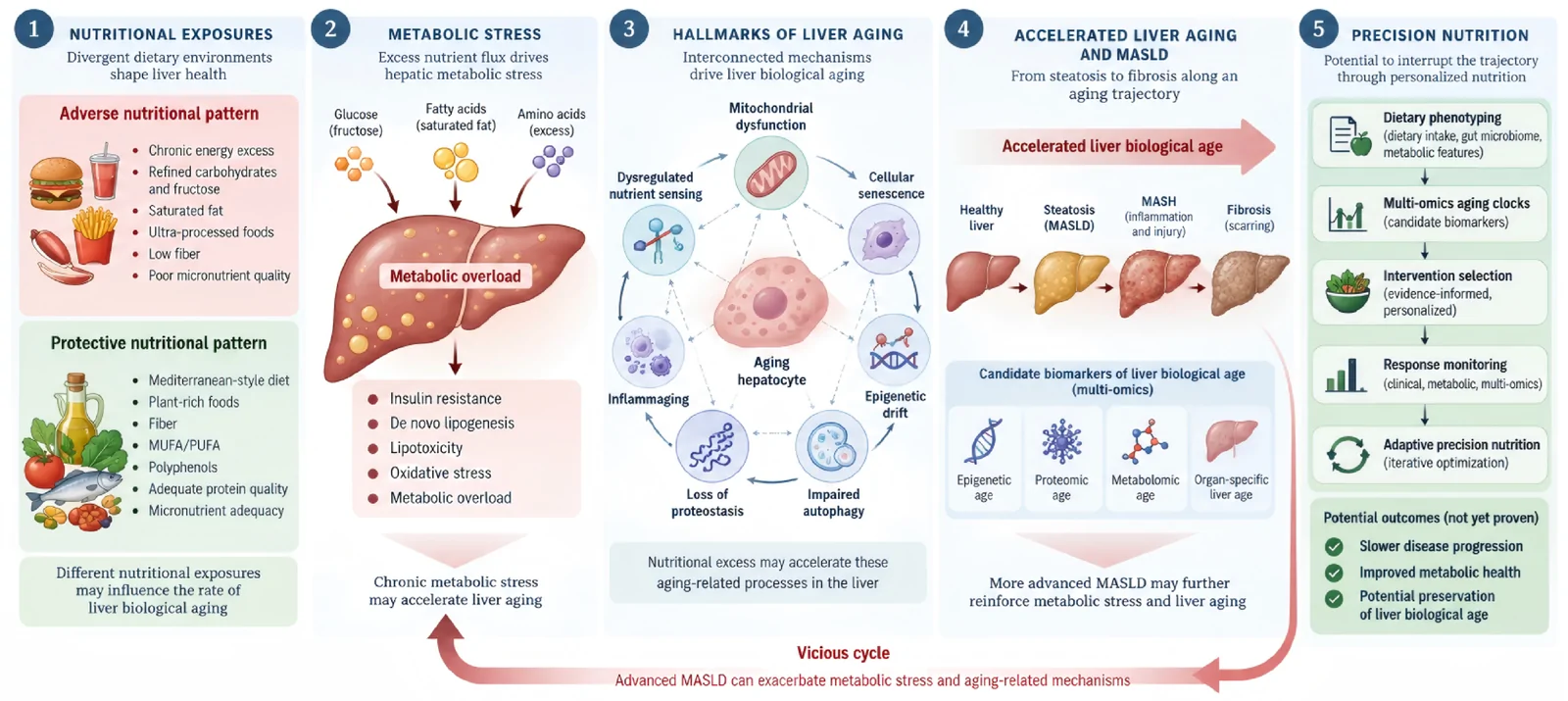
Nutrition, liver aging, and the MASLD trajectory. The schematic links nutritional exposures to metabolic stress, aging-related mechanisms, and progressive liver injury. Arrows summarize biologically plausible connections, not a single experimentally established causal sequence. MASLD can progress, remain stable, or regress; the depicted sequence is not inevitable. Precision nutrition is shown as a potential intervention point, whereas prevention of liver aging remains an investigational outcome. MASLD, metabolic dysfunction-associated steatotic liver disease; MASH, metabolic dysfunction-associated steatohepatitis; MUFA, monounsaturated fatty acids; PUFA, polyunsaturated fatty acids.

Nutritional benefit can follow at least two overlapping routes. Dietary change can reduce adiposity and thereby alter metabolic stress and liver outcomes; it can also modify substrate handling, lipid composition, or microbial signaling through pathways not fully mediated by weight change. The total treatment effect includes both routes. An effect estimated after weight-loss adjustment addresses a different question and is not inherently more informative. Baseline adiposity may confound an observational association, whereas weight change after randomization may mediate an intervention effect. Figure 1 places these routes in their biological context; subsequent sections evaluate their evidence separately.

## 2. Review Scope and Literature Selection

This critical narrative review integrated searches of PubMed/MEDLINE, Web of Science Core Collection, Scopus, and Google Scholar conducted from 1 June to 15 September 2026, with citation chasing of pivotal guidance and primary studies through 30 September 2026. Four complementary domains organized retrieval: MASLD and biological aging; nutrition and aging measures; nutritional intervention, MASLD, and molecular profiling; and MASLD and precision nutrition. The core concepts were combined as (MASLD OR NAFLD OR NASH OR “metabolic dysfunction-associated fatty liver disease”) AND (nutrition OR diet OR dietary OR weight loss OR fiber OR Mediterranean OR metabolom* OR epigen* OR proteom* OR biological aging OR senescence OR clock OR fibrosis). Database-specific strings, eligibility rules, approximate record counts, and the screening audit trail are provided in Appendices Appendix A and Appendix B. Searches included historical terms only to identify the original disease definition; recommendations are framed for contemporary MASLD.

Reports were included when they informed at least one of the following: a human MASLD–aging association; a nutritional intervention with hepatic or aging outcomes; validation of an aging measure; a specific nutritional mechanism relevant to hepatic injury; or a clinical decision concerning fibrosis, metabolic risk, or nutritional adequacy. Recent primary studies, particularly those published from 2021 onward, were prioritized alongside clinical guidelines. Earlier work was retained for foundational measures and pivotal mechanistic or clinical evidence. Reports without a relevant exposure, outcome, measurement method, or clinical implication were excluded from the synthesis. Duplicate reports were consolidated using publication identifiers; companion analyses were linked conceptually to their parent trials to avoid treating biomarker subsets as separate randomized populations. One author screened titles/abstracts and full texts against the prespecified rules, and a second author independently checked all included studies and all exclusions judged uncertain. Disagreements were resolved by discussion with the senior author before extraction.

Selection emphasized relevance and methodological informativeness, including studies that challenged an apparent benefit. Appraisal considered randomization and comparator quality, attrition, analysis population, exposure measurement, confounding, temporal ordering, multiplicity, tissue specificity, external validation, and outcome relevance. These domains informed interpretation rather than a numerical quality score. Experimental models were used for mechanism, observational human studies for association or prognosis, and randomized studies for intervention effects. Narrative selection and domain-based appraisal support a critical synthesis; they do not provide the exhaustiveness or pooled inference of a systematic review.

### Study Design and Tissue Source Determine Evidence Directness

Direct evidence for the central question requires a nutritional intervention in a defined MASLD population together with a prespecified aging endpoint. Within that category, a liver-tissue or independently validated liver-specific endpoint is more informative than a systemic clock. The studies reviewed do not establish this complete chain. Indirect hepatic evidence measures liver outcomes after nutritional treatment without an aging endpoint; extrapolated aging evidence measures nutritional effects on aging biomarkers in populations not selected for MASLD. MASLD association and prognosis studies, preclinical mechanisms, and pharmacological comparisons provide complementary context. These categories describe directness to the question, not a substitute for study quality: a rigorous trial outside MASLD can have strong internal validity while remaining indirect for hepatic aging. Table 1, Table 2, Table 3, Table 4 and Table 5 identify these distinctions alongside the original designs.

## 3. Aging Associations in MASLD Depend on Tissue and Disease Stage

### 3.1. Tissue and Systemic Measures Capture Different Components of Disease Burden

The strongest early demonstration of tissue specificity came from Horvath and colleagues: higher body mass index was associated with epigenetic age acceleration in liver, but not consistently in blood, muscle, or adipose tissue [10]. The estimated difference was 3.3 years per 10 body mass index units. Importantly, hepatic age acceleration was not associated with the NAFLD activity score after adjustment for body mass index and did not rapidly reverse following bariatric weight loss. The study established a liver-specific association with obesity, while cautioning against interpreting short-term weight loss as reversal of liver-specific aging.

Peripheral blood can nevertheless carry information about hepatic injury. In biopsy-proven NASH with stage F2–F3 fibrosis, Loomba and colleagues found accelerated DNA methylation (DNAm) age that correlated with hepatic collagen content, although it did not distinguish the included fibrosis stages [11]. In the Shanghai Changfeng Study, Xia and colleagues linked baseline methylation age acceleration to subsequent increases in ultrasound-estimated liver fat [12]. The adjusted odds ratio comparing the highest with the lowest age-acceleration tertile was 4.55, but its confidence interval was wide (1.06–19.61). These studies support a biological signal while leaving its incremental clinical discrimination uncertain.

The relationship is not uniform across disease definitions. Tong and colleagues analyzed 3974 National Health and Nutrition Examination Survey (NHANES) participants and found that advanced fibrosis, but not MASLD itself, was independently associated with older clinical biological-age estimates [13]. A separate two-cohort study in the United Kingdom and China associated accelerated biological aging with incident NAFLD without a significant interaction with genetic susceptibility [54]. These findings can coexist: a systemic age measure may identify upstream metabolic risk, whereas its cross-sectional association with existing steatosis can weaken after adjustment for the same metabolic factors used to define disease.

Prognostic associations strengthen the rationale for investigation. Among 2199 NHANES participants with NAFLD followed for a median of 16 years, higher clinical biological-age measures were associated with mortality [55]. This does not demonstrate that lowering an age estimate lowers risk, but it identifies an outcome against which a candidate biomarker can be evaluated. In chronic liver disease, simultaneous acceleration of skeletal muscle and immune aging further suggests that the relevant vulnerability extends beyond the liver [56]. For nutritional care, this systemic component matters because an intervention that improves steatosis while worsening muscle function may be clinically unsuccessful.

### 3.2. Fibrosis Exposes Differences Between Clocks

A 2025 analysis of 325 individuals provides a particularly informative comparison. Advanced fibrosis was associated with higher DunedinPACE and lower DNAm-estimated telomere-length acceleration, whereas intrinsic epigenetic age acceleration, PhenoAge acceleration, and GrimAge acceleration were not significantly associated [14]. The DunedinPACE coefficient was 0.05 (95% confidence interval, 0.03–0.07). This result is best interpreted on the pace scale; it is not equivalent to five additional years of liver age. It also argues against choosing a clock solely because it is widely available.

Direct liver tissue analysis offers a different perspective. Van Dijck and colleagues studied 22 biopsies and found progressive methylation changes across MASLD stages [15]. Horvath age acceleration tracked several histological features, LiverClock tracked disease stage, and HepClock and intrinsic age acceleration did not show the same associations. Integration with a larger expression dataset implicated redox, immune, and metabolic regulatory networks. The small biopsy sample makes the findings mechanistically informative, but insufficient to rank these clocks for clinical use.

The 2026 multicenter study of MASLD-related hepatocellular carcinoma included 272 cases and 316 cancer-free MASLD controls. Associations were present for principal-component PhenoAge and GrimAge and for DunedinPACE, but not for principal-component HorvathAge or HannumAge [17]. Because the comparison was case–control, it cannot establish that these measures predict cancer before diagnosis. Nor should it alter established surveillance pathways. Together, the studies in Table 1 support a stage- and measure-dependent aging phenotype rather than the blanket classification of every patient with MASLD as biologically older.

## 4. Nutritional Stress Disrupts Pathways of Hepatic Maintenance

Nutritional stress connects liver disease with aging through interacting defects in substrate handling, cellular repair, and tissue communication. The mechanisms reviewed below explain why liver fat alone cannot capture the full response to dietary change. They also identify a prevention-relevant research priority: reducing hepatic injury while preserving metabolic and physical function across adulthood.

### 4.1. Nutrient Sensing and Mitochondrial Adaptation Shape Susceptibility

Persistent nutrient surplus requires the liver to coordinate substrate oxidation, storage, export, and repair. In experimental NASH, reduced DEAD-box protein 5 activity disrupts tuberous sclerosis complex regulation of mechanistic target of rapamycin complex 1 (mTORC1). Restoring this interaction improves lipid handling and reduces inflammatory signaling [57]. This mechanism links nutrient sensing to injury, but its response to dietary change remains to be established. Translation also requires balancing hepatic effects against the roles of nutrient signaling in other tissues.

Age modifies the hepatic response to the same nutritional challenge. In a 2026 comparison of young and old male mice exposed to a high-fat diet, older animals showed more severe steatosis, inflammation, and transcriptional disturbance. Rapamycin reversed many diet-associated transcriptional changes, supporting age-dependent sensitivity to nutritional stress [58]. Translation to people requires attention to sex, immune effects, and skeletal-muscle maintenance. The experiment does not establish rapamycin as a nutritional or clinical strategy for MASLD.

Human studies place mitochondrial adaptation, rather than respiratory activity alone, at the center of interpretation. Respiratory capacity was enhanced in obesity with or without simple steatosis, whereas this adaptation was lost in NASH [59]. A later biopsy study found lower oxidative capacity in NASH with type 2 diabetes than in NASH without diabetes, despite comparable hepatic fat and histology [60]. Liver triglyceride content and mitochondrial resilience therefore represent related but distinct outcomes. Dietary-response studies should account for diabetes and fibrosis when interpreting metabolomic changes.

Nicotinamide adenine dinucleotide (NAD^+^) links redox metabolism with cellular repair and provides another candidate intervention target. A critical review of NAD^+^ precursors found broad experimental effects but limited, heterogeneous human evidence [61]. Inhibition of alpha-amino-beta-carboxymuconate-epsilon-semialdehyde decarboxylase enhanced hepatic de novo NAD^+^ synthesis and reduced DNA damage, inflammation, and fibrosis in mouse and human liver-organoid models [62]. These findings support a liver-directed therapeutic hypothesis that requires clinical testing. Evidence that commercially available precursors slow hepatic aging remains insufficient; future trials need target-engagement measures and liver outcomes before preventive use can be supported.

### 4.2. Cell Identity Determines the Consequences of Senescence

Hepatocyte senescence can impair fatty-acid metabolism and sustain inflammatory signaling. Genetic clearance and senolytic treatment reduced steatosis in mice, while experimentally induced hepatocyte senescence promoted lipid accumulation [63]. Human biopsies provide complementary observational evidence: hepatocyte p21 expression and nuclear enlargement were associated with fibrosis and adverse liver-related outcomes. Telomere length was less informative for prognosis in that study [64]. In young mice, diet-associated senescence developed without telomere shortening or global methylation changes [65]. Metabolic stress can therefore activate this pathway before advanced chronological age, providing a rationale for studying prevention across adulthood.

The consequences of senescence differ across hepatic cell populations and disease processes. Senescence of activated hepatic stellate cells can limit fibrogenesis by arresting proliferation and facilitating immune clearance [66]. A general aim to “reduce senescence” therefore conceals potentially opposing effects. Cell-resolved measurements are needed to distinguish improved hepatocyte function, arrested stellate-cell activation, and altered immune surveillance. A blood-clock response can complement these observations, but cannot identify the affected cell type or establish the direction of tissue benefit.

### 4.3. Lipid Processing and Epigenetic Regulation Connect Diet with Tissue Injury

Hepatic lipid accumulation reflects coordinated failures in disposal, export, and cellular maintenance. Inhibition of autophagy increased triglyceride storage in hepatocytes and mouse liver, implicating lipophagy in intracellular lipid disposal [67]. Disturbed phospholipid synthesis and lipoprotein secretion following loss of vacuole membrane protein 1 produced severe steatosis, inflammation, and fibrosis [68]. These mechanisms place organelle structure and lysosomal processing alongside substrate oxidation when evaluating a nutritional response.

Membrane lipid composition further separates fat quantity from its biological consequences. Loss of hepatic MBOAT7 promoted fibrosis with relatively limited inflammatory change, implicating phosphatidylinositol remodeling in an inflammation-independent fibrogenic pathway [69]. Loss of lysophosphatidylcholine acyltransferase 3 increased mitochondrial membrane saturation and disrupted mitochondrial homeostasis; restoring expression ameliorated experimental NASH [70]. Equal reductions in total liver fat may therefore conceal different changes in lipotoxic species and tissue injury. This distinction supports evaluating nutritional effects with complementary measures of liver fat and fibrosis risk.

Dietary effects on epigenetic regulation provide a potential connection between exposure and tissue-relevant molecular information. In an 18-participant study, Mediterranean-style dietary modification with fiber supplementation altered blood methylation patterns. The identified sites also stratified fibrosis in independent liver-biopsy data [71]. This small study supports further evaluation of blood-based molecular responses to diet. A fibrosis classifier nevertheless measures disease-related variation; its status as an aging measure or marker of treatment benefit requires separate longitudinal validation.

### 4.4. Immune and Gut–Liver Signaling Modify Injury Beyond Weight Change

Immune signaling combines pathways of injury with compensatory and protective responses. Myeloid-specific inactivation of brain-abundant membrane-attached signal protein 1 reduced inflammasome activation and fibrosis in mice [72]. By contrast, macrophage-derived osteopontin protected against experimental NASH through oncostatin-M, STAT3, and hepatocyte arginase-2 signaling [73]. Neutrophil extracellular traps provide another route to fibrosis by driving the metabolic activation of stellate cells [74]. An aggregate inflammatory protein score can combine these different processes, limiting its interpretation as a direct measure of hepatic improvement.

Dietary modulation of the microbiota provides a route to hepatic effects beyond weight change. High dietary cholesterol promoted liver cancer in mice through changes in microbial communities and metabolites; transfer experiments supported a contribution from the microbiota [75]. Oat beta-glucan reduced inflammation and fibrosis in another dietary mouse model without substantially changing weight gain or glucose intolerance. Antibiotic-mediated microbiota disruption abolished this benefit, supporting a microbiota-dependent effect in that model [76]. The findings justify testing dietary composition alongside energy balance, with clinical effectiveness established separately for each intervention.

Mechanistic synthesis connects microbial metabolites with mitochondrial redox dysfunction and ferroptosis-sensitive injury across cardiometabolic diseases, including MASLD [77]. Evidence for individual links in this pathway should be distinguished from an experimentally complete causal chain. Nutritional studies can address this gap by assessing lipid peroxidation, antioxidant defense, and tissue injury in the relevant compartment. A circulating oxidative-stress marker or a lower aging score alone provides limited evidence that hepatic ferroptosis has changed. Redox biology helps select mechanistic endpoints, while liver outcomes remain necessary to establish benefit.

Cross-organ and food-system transcriptomic studies further illustrate why redox and lipid programs should be treated as mechanistic context rather than liver-specific endpoints. In proximal-tubule injury, ergothioneine-associated transcription opposed conserved mitochondrial injury programs involving oxidative phosphorylation, respiratory electron transport, complex I biogenesis, and mitochondrial translation [78]. In a separate cross-dataset analysis of *Aspergillus flavus*, an expression-defined redox–lipid state was associated with relative aflatoxin biosynthetic activity across food-relevant conditions [79]. These studies are not MASLD interventions and do not establish hepatic benefit; they support a hypothesis-generating strategy that measures redox state, lipid remodeling, transcriptional response, and direct tissue outcomes together.

Model selection and participant biology constrain the translation of these mechanisms. An evaluation of murine MASLD models found that Western diets more closely approximated human MASH. Choline-deficient models rapidly produced fibrosis but had poorer metabolic translatability [80]. Sex also changed dietary responses and the hepatic PPAR-alpha regulatory network in an integrative mouse–human analysis [81]. Mechanisms inferred from a single male, nutrient-deficient model provide experimental leads with limited support for universal dietary prescriptions.

Together, these pathways support a broader interpretation of nutritional prevention than reduction of liver fat alone. The translational task is to establish which sustainable dietary changes reduce tissue injury while maintaining function, and which measurements can track those benefits reliably. Mechanistic detail strengthens that task when it explains heterogeneity, selects informative outcomes, or identifies a testable intervention target.

## 5. Aging Measures Capture Distinct Dimensions of Risk and Response

The public health value of an aging measure depends on the information it adds to prevention and care. A useful measure should improve risk assessment, clarify treatment response, or support evaluation of sustained health benefit. These roles require different evidence, and none follows automatically from accurate prediction of chronological age.

### 5.1. Training Targets Define the Interpretation of Aging Measures

“Biological age” can refer to chronological-age prediction, a mortality-calibrated score, or the estimated pace of physiological decline. These quantities differ in their units, intended uses, and interpretation of change. Age acceleration commonly means the residual from regressing predicted age on chronological age within an appropriate reference population. The unadjusted difference between predicted and chronological age is sensitive to calibration and regression toward the mean. DunedinPACE instead estimates the pace of multisystem aging and should not be reported as years of age.

First-generation clocks demonstrate the distinction between age prediction and clinical response. Horvath’s multi-tissue model estimates age across human tissues and cell types [31]. Hannum’s blood-based model captures age-related methylation patterns in a more specific biological compartment [30]. Neither was optimized to detect nutritional response or predict fibrosis regression. A small change in chronological-age prediction error therefore requires additional validation before it can indicate treatment benefit.

Health-related training targets shift the emphasis toward variation associated with adverse outcomes. DNAm PhenoAge used a two-stage construction linking methylation to a clinical phenotype predictive of mortality [29]. GrimAge incorporated methylation surrogates for circulating proteins and smoking exposure and showed strong associations with morbidity and mortality [32]. Both remain epigenetic measures despite the clinical or protein-related information used in their construction. Clinical PhenoAge is calculated directly from blood chemistry, whereas proteomic clocks measure proteins themselves; these distinctions matter when attributing a dietary response.

DunedinPACE was trained against longitudinal change in 19 physiological indicators over two decades within a birth cohort [33]. Its sensitivity to nutritional intervention may partly reflect this emphasis on pace rather than accumulated age. As a blood-based measure of systemic aging, it cannot acquire liver specificity from an association with advanced fibrosis alone. Table 3 compares the major classes by intended use and the evidence still needed for MASLD.

### 5.2. Clinical Measures Anchor Evaluation in Accessible Risk Assessment

Routine clinical biological-age measures cost less than omics assays and can be applied to large cohorts. This makes them relevant comparators for population studies and evaluations of more complex tests. Their interpretation requires attention to metabolic and inflammatory components directly affected by diet and disease. Principal-component clinical clocks such as PCAge and its streamlined derivatives identified metabolic, inflammatory, cardiac, and renal patterns associated with adverse aging. A tailored CALERIE analysis detected improvement during calorie restriction [82]. Such composite improvement may summarize useful change without identifying a process independent of the component biomarkers.

The 2026 Liver Aging Index extends this approach toward hepatology. Developed in 21,629 China Kadoorie Biobank participants, it combined clinical variables, eight plasma biomarkers, and liver fat and stiffness measurements. Validation included NHANES and a 16-center prognostic cohort, with reported discrimination for all-cause mortality of approximately 0.76 in the external cohorts [16]. Its use of clinical measurements and explicit linkage to liver-related outcomes supports further translational evaluation. Because liver stiffness, liver enzymes, lipids, and glucose contribute to the index, diet-induced improvement can lower the score by construction. Incremental prediction beyond these components is therefore the decisive clinical comparison. For wider implementation, any added predictive value also needs to justify the availability and cost of the required assays and imaging.

### 5.3. Proteomic Clocks Resolve Heterogeneity but Infer Organ Specificity

Organ-enriched plasma proteins offer a way to examine heterogeneity in aging across the body. Models estimating aging across 11 organs were validated in five cohorts encompassing 5676 adults [38]. These estimates indicated that marked age acceleration can be concentrated in one organ. Organ enrichment, however, is a biological annotation; circulating abundance also depends on secretion, tissue damage, distribution, and clearance. Interpretation of a liver-related score therefore requires evidence beyond the proteins’ tissue annotations.

Large studies establish broad prognostic relevance while leaving nutritional responsiveness open. A 204-protein age clock developed in 45,441 UK Biobank participants showed associations with 18 incident diseases, with age prediction validated in Chinese and Finnish populations [34]. Subsequent work combined conventional and organ-specific models trained on age, mortality, and longitudinal proteomic data in more than 50,000 participants [36]. The resulting diet and lifestyle associations support hypothesis generation, but cannot establish that changing a dietary exposure slows organ aging.

Further organ-specific models developed in 43,616 UK Biobank participants were validated in Chinese and US cohorts. They predicted disease and mortality beyond selected clinical and genetic variables [37]. An independent 2026 evaluation in the European Prospective Investigation into Cancer and Nutrition, with Whitehall II replication, extended follow-up and disease coverage. Mortality prediction was nevertheless comparable to classical lifestyle risk factors [35]. This comparison makes added decision value central to implementation: molecular detail should improve choices beyond risk already evident from smoking, adiposity, and physical inactivity. Platform differences, training populations, and model construction further constrain transfer across settings [83]. Evaluation should therefore establish whether proteomic testing improves prevention sufficiently to justify its additional cost and complexity.

### 5.4. Metabolomic Clocks Detect Nutritional Variation and Require Standardized Sampling

Metabolomics measures substrates and products involved in hepatic metabolism, making it attractive for nutritional-response research. A UK Biobank study trained age estimates from 168 metabolites and evaluated 50 incident diseases; associations persisted for 15 diseases after full adjustment [25]. A separate mortality-trained model, MetaboAgeMort, used 239,291 participants and identified strong associations with body composition, smoking, socioeconomic conditions, and dietary quality [26]. These associations support a broad population health perspective on metabolic variation. Liver-related genetic enrichment nevertheless requires further validation before the model can be interpreted as a liver-specific assay.

Training targets materially change the interpretation of diet and lifestyle associations. Across three prospective cohorts totaling 24,332 participants, adherence to dietary and physical activity guidelines was inversely associated with MetaboHealth. MetaboAge showed a different pattern of associations in the same analysis [27]. A smaller study linked a plasma signature of decelerated physiological aging, including tricarboxylic-acid-cycle metabolites, to an accessible physiological score [28]. These constructs describe different aspects of physiology and should retain their separate definitions when used in MASLD research.

Metabolites can change rapidly after a meal, fasting, weight loss, or medication. A decline in a metabolomic age estimate may sensitively register exposure or response without demonstrating durable tissue repair. Interpretation also depends on platform coverage and the biological target used to construct the clock [84]. In MASLD, fasting duration, sampling time, and recent exercise should be standardized, with renal function and medication exposure recorded and considered analytically. These procedures help separate a sustained treatment response from transient metabolic state.

Physical and cognitive function provide external reference points for the relevance of metabolic changes to healthy aging. An observational analysis across cognitive aging stages derived a lifestyle-associated blood metabolic pathway score related to physical and cognitive performance and frailty [85]. Pathway-domain patterns were more stable than individual pathway coefficients in internal analyses. This non-MASLD study supports examining functional relevance, while leaving longitudinal responsiveness and independent validation open. The score summarizes lifestyle-associated metabolic variation and should be distinguished from an aging clock or liver-specific measure. In nutritional MASLD studies, functional outcomes could help determine whether molecular improvement accompanies benefits that matter in daily life.

Table 2 organizes the metabolomics evidence by intended application. These roles are deliberately separated: an exposure marker documents intake or adherence, an association biomarker relates to disease, a prognostic signature estimates future risk, a response biomarker changes after an intervention, and a candidate surrogate endpoint would need to predict later clinical benefit across interventions. None of the metabolomic aging scores currently satisfies the last criterion in MASLD.

### 5.5. Integration Gains Value Through Complementarity and Clinical Validation

**Figure 2:**
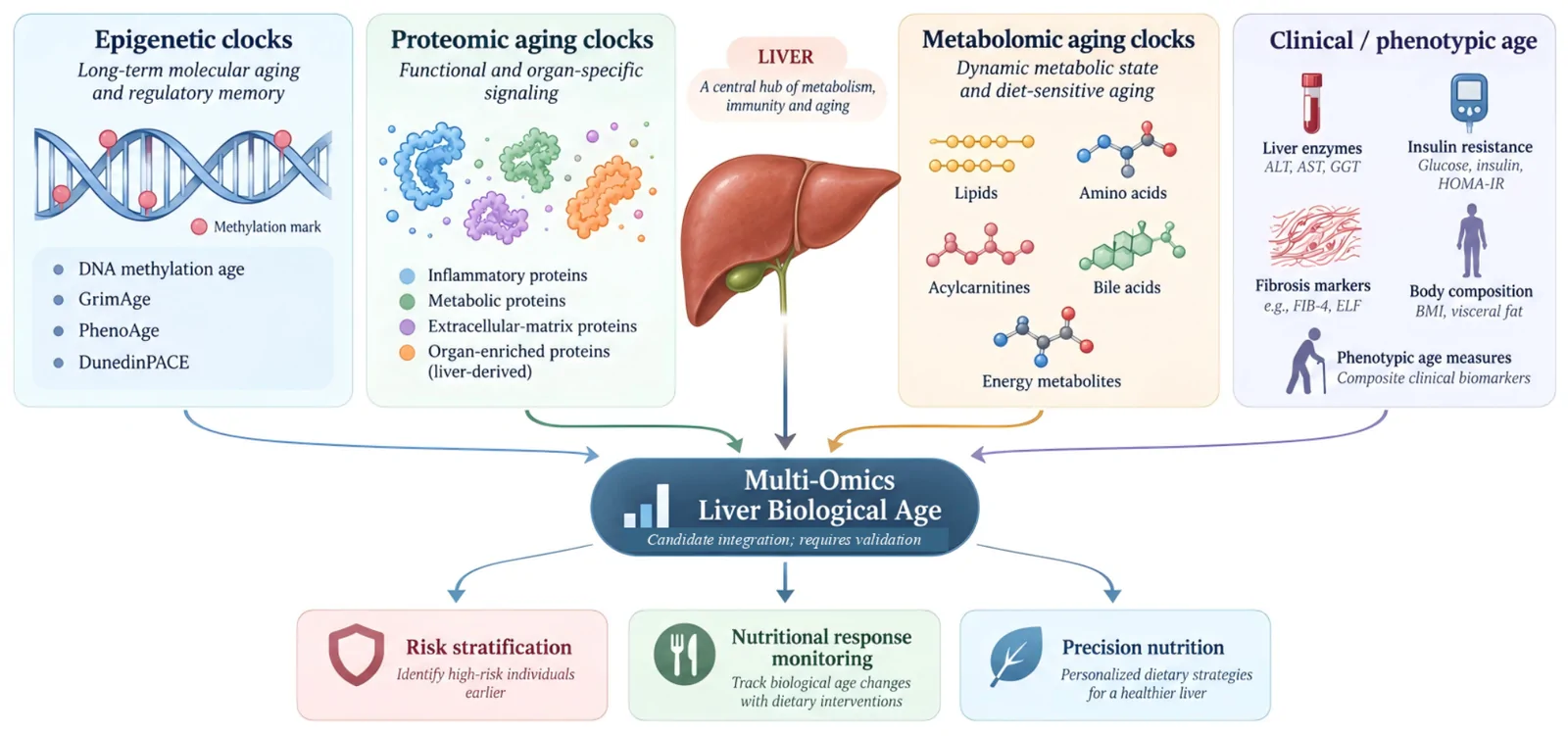
Investigational multi-omics framework for liver-aging research. Epigenetic, proteomic, metabolomic, and clinical measurements provide complementary information. Their integration and the downstream applications shown remain investigational and are not a validated clinical approach. Circulating organ-enriched proteins are not direct liver-tissue measurements, and clinical variables such as liver enzymes, insulin resistance, and fibrosis scores are not standalone aging clocks. DNAm PhenoAge should be distinguished from clinical PhenoAge. ALT, alanine aminotransferase; AST, aspartate aminotransferase; GGT, gamma-glutamyl transferase; HOMA-IR, homeostatic model assessment of insulin resistance; FIB-4, Fibrosis-4 index; ELF, Enhanced Liver Fibrosis test; BMI, body mass index.

Figure 2 presents an investigational framework for jointly assessing epigenetic, proteomic, metabolomic, and clinical information. Its central “Investigational multi-omics liver-aging framework” label denotes a research hypothesis that remains to be validated as a test. The intended advantage is complementary information: methylation may reflect accumulated regulation, proteins functional signaling, and metabolites current substrate handling. Combining these measurements into one score can obscure informative disagreement between biological domains. A decrease in a metabolomic aging estimate with unchanged epigenetic pace may indicate rapid metabolic adaptation. Separate reporting retains this distinction and helps determine which measures add useful information.

The three inferential steps introduced earlier define the evidence needed for such integration. Responsiveness means that a measure changes reproducibly after intervention under standardized sampling. Modification of an aging-related process requires evidence of persistent biology beyond acute substrate exposure, cell-composition shifts, or assay variation. Surrogate validity requires treatment-induced changes to predict later clinical benefit across suitable interventions and populations. Nutritional sensitivity can satisfy the first step while leaving the second and third unresolved. Additional omics layers provide information for testing these requirements but cannot substitute for longitudinal and interventional validation.

Methodological studies show why each inferential step requires its own evidence. Accumulating stochastic variation can generate accurate aging clocks, separating age prediction from identification of a causal aging program [86]. Experiments in primary human cells also distinguished epigenetic aging from several other hallmarks, including cellular senescence and telomere attrition [87]. Together, these studies support interpreting each construct on its own terms and using complementary biological measurements to assess mechanistic claims.

Reliable measurement and a defined intended use are prerequisites for individual monitoring. Principal-component versions of epigenetic clocks substantially reduced replicate variability in a dedicated benchmarking study [88]. TIME-seq offers a route to lower-cost methylation measurement, although each clock still requires validation in its intended population and for longitudinal use [89]. The nutrition-biomarker perspective similarly emphasizes matching the biomarker to a defined research question before implementation [39]. A prespecified panel with distinct roles currently provides a more interpretable research strategy than an unvalidated composite “multi-omics liver age.”

For population implementation, evaluation should also establish affordability, calibration across population groups, and added benefit over accessible clinical assessment. A practical research design can pair clinical and functional outcomes with selected molecular measures whose contribution is prespecified. This approach makes the purpose of measurement explicit: improving prevention decisions and demonstrating sustained health benefit.

## 6. Diet Quality and Nutrient Adequacy Shape Cumulative Metabolic Burden

### 6.1. Energy Balance and Food Quality Provide Complementary Prevention Targets

Sustained excess energy, food composition, and eating behavior jointly shape nutritional stress. In Hall and colleagues’ inpatient crossover trial, an ultra-processed diet increased ad libitum energy intake and body weight relative to an unprocessed diet [90]. The experiment identifies a behavioral route through which everyday food choices can increase metabolic burden. In Moli-sani, higher ultra-processed food intake was associated with a small increase in clinical biological age after accounting for Mediterranean diet quality [91]. Residual differences in socioeconomic circumstances and health remain plausible explanations for part of that association. Together, these findings support considering the food environment alongside individual dietary advice, while evidence for modifying hepatic aging remains indirect.

A processing-centered gut–liver–brain review proposes that MASLD may amplify metabolic and inflammatory signals associated with ultra-processed food exposure, with possible implications for cognitive aging [92]. Its framework distinguishes nutrient composition from food-matrix disruption, eating rate, and displacement of microbial substrates. These proposed pathways support recording processing alongside energy and nutrient intake in nutrition studies. They also broaden the outcomes worth investigating beyond steatosis, although cognitive and hepatic aging benefits require direct testing.

Cumulative exposure gives dietary prevention a life-course rationale. In young adults followed from childhood, persistent obesity was associated with a broader pattern of inflammatory, nutrient-sensing, and aging-related biomarkers [93]. A single body mass index measurement therefore provides an incomplete account of the exposure history relevant to these pathways. This evidence supports earlier attention to sustained dietary quality and excess adiposity, while the effects of specific preventive programs require prospective evaluation.

Carbohydrate quality offers a specific target within this broader prevention strategy. In a seven-week randomized study of healthy men, fructose- and sucrose-sweetened beverages increased basal hepatic fatty-acid synthesis; glucose-sweetened beverages did not show the same effect [94]. The finding concerns beverage exposure and lipogenesis, so it cannot be generalized to whole fruit or fibrosis progression. A LifeLines-DEEP analysis related higher dietary glycemic load, food processing, and poorer micronutrient adequacy to several epigenetic measures [95]. The practical priority is to reduce sugar-sweetened beverages and improve carbohydrate sources while maintaining nutritional adequacy. Evaluation of population programs should establish whether affordable substitutions produce sustained changes in intake and established metabolic outcomes.

### 6.2. Fat Substitution and Protein Adequacy Support Distinct Treatment Goals

The source of excess energy changes the hepatic metabolic response. An overfeeding experiment in 38 adults found a 55% increase in intrahepatic triglycerides with saturated fat, compared with 15% with unsaturated fat [96]. Simple sugars increased liver fat primarily through de novo lipogenesis (DNL), whereas saturated fat also increased insulin resistance and several ceramides. These findings support substituting unsaturated for saturated fats within an appropriate energy intake. Adding energy-dense foods without substitution would not reproduce that nutritional strategy.

Metabolomic profiling can connect dietary exposure with changes in substrate handling. A comparison of gas chromatography, nuclear magnetic resonance, and liquid chromatography–mass spectrometry found platform-dependent agreement for dietary fatty-acid biomarkers [18]. Assay selection therefore affects the comparability of adherence and response measurements. Changes in a lipid-containing metabolomic clock may also partly reflect the altered dietary exposure itself. Analyses should distinguish exposure confirmation, metabolic response, and aging assessment so that rapid substrate shifts are not mistaken for durable restoration of hepatic resilience.

Protein adequacy makes functional preservation a central goal of nutritional care. Amino-acid signaling is relevant to nutrient sensing, but experimental antiaging models do not establish safe protein-restriction targets for older adults with MASLD. The AASLD guidance on malnutrition, frailty, and sarcopenia in cirrhosis prioritizes adequate protein and avoidance of prolonged fasting [97]. During energy restriction in noncirrhotic MASLD, assessment of muscle function, dietary protein adequacy, and physical activity helps address competing treatment needs. Reducing excess adiposity while preserving functional reserve is particularly relevant to healthy aging and continued independence.

A prospective analysis of aging Chinese adults reported reciprocal associations between diabetes and sarcopenia after accounting for time spent in each disease state [98]. Although the cohort was not selected for MASLD, it supports considering glycemic status and muscle health together during follow-up. Its counterfactual estimates model preventable progression under specified assumptions; they are not observed effects of dietary treatment. Strength and physical function therefore belong alongside metabolic outcomes when assessing the value of weight-reduction programs for older populations.

### 6.3. Fiber and Micronutrient Adequacy Strengthen a Food-Based Approach

Fiber interventions provide human evidence that dietary composition can improve steatosis and modify microbial metabolism. A four-month randomized trial analyzed 196 participants receiving resistant starch or control [22]. The absolute reduction in intrahepatic triglyceride content versus control was 9.08 percentage points and remained 5.89 points after adjustment for weight loss. Reductions in circulating branched-chain amino acids and specific microbial taxa accompanied the liver response; transfer and colonization experiments in mice supported mechanistic involvement. This trial establishes benefit for a specific fiber intervention in NAFLD, with the weight-adjusted estimate addressing a different effect from the total dietary response.

A smaller, uncontrolled study in 27 patients replacing bread with high-fiber buns reported reduced controlled attenuation parameter (CAP) and altered serum metabolites [23]. Some circulating short-chain fatty acids decreased, illustrating why one peripheral metabolite cannot define improved fermentation or hepatic health. The food substitution also provides a useful implementation model for research on familiar staple foods. Larger controlled studies should assess affordability, acceptability, and persistence alongside efficacy before such substitutions are incorporated into broader programs.

One-carbon nutrients connect dietary adequacy, methyl-group availability, and protein homeostasis. Experimental work linked hyperhomocysteinemia to homocysteinylation of syntaxin 17, impaired autophagy, and NASH [99]. Vitamin B12 and folate supplementation restored autophagy and improved established disease in mice. In 2346 older NHANES participants, folate and homocysteine showed opposing associations with several epigenetic aging measures [100]. Renal function and smoking materially influenced the observed age associations. The translational priority is adequate intake and correction of documented deficiency; neither the experimental mechanism nor the observational associations establish a high-dose clock-directed treatment.

**Figure 3:**
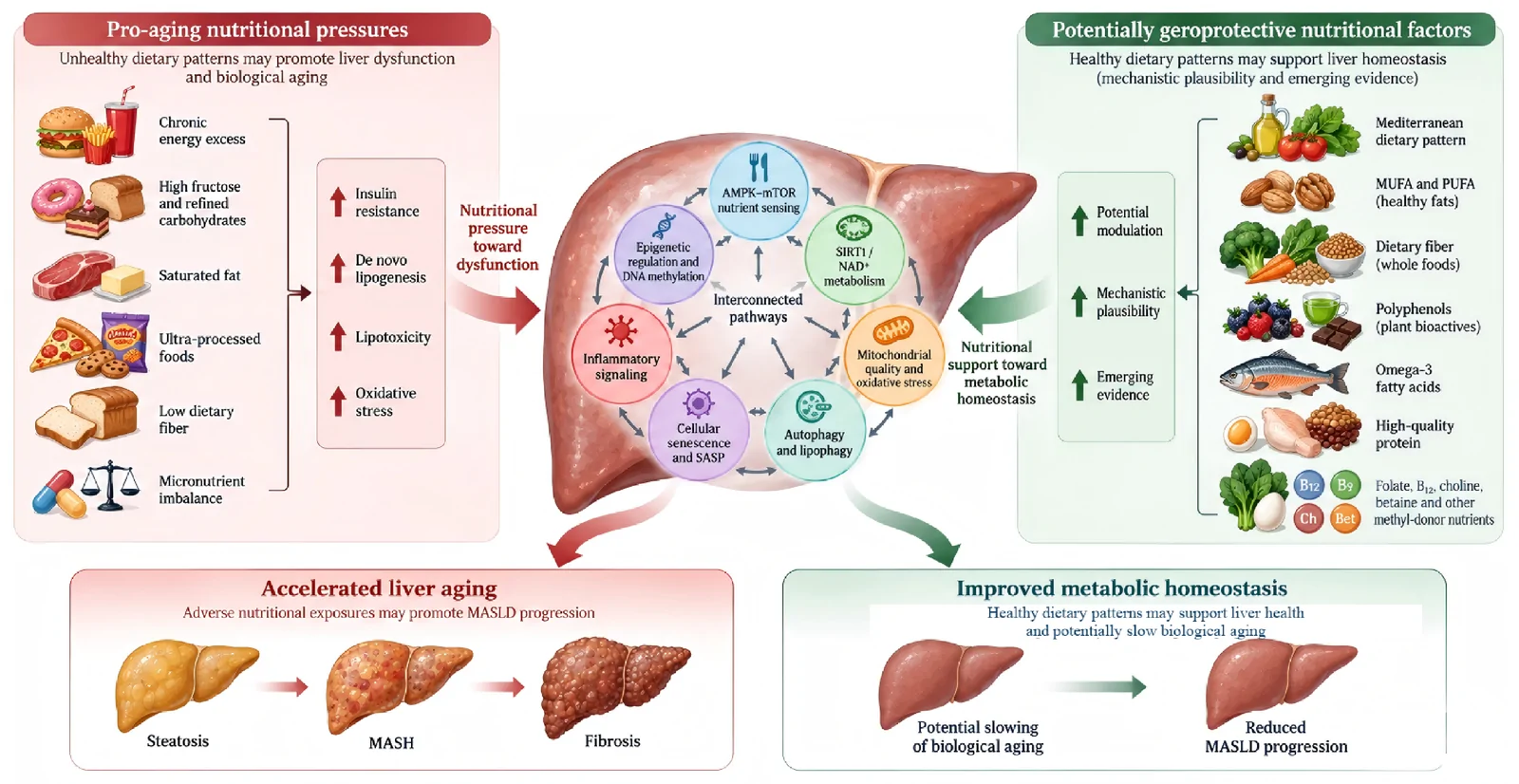
Nutritional modulation of liver aging-related pathways. The diagram summarizes proposed links between dietary exposures, metabolic stress, nutrient sensing, mitochondrial quality, autophagy, inflammation, and epigenetic regulation. Evidence ranges from human feeding studies to experimental models; effects on liver fat should not be equated with proven slowing of liver aging or fibrosis progression. Protein and micronutrient adequacy require individual assessment. AMPK, AMP-activated protein kinase; mTOR, mechanistic target of rapamycin; SIRT1, sirtuin 1; NAD^+^, nicotinamide adenine dinucleotide; SASP, senescence-associated secretory phenotype.

Plant bioactives are best evaluated within the foods and dietary patterns that deliver them. A 2026 study in old mice linked an anthocyanin to expansion of *Limosilactobacillus reuteri*, altered bile-acid metabolism, and hepatic farnesoid X receptor signaling [101]. Murine bile-acid biology and dependence on microbial metabolism limit direct dose translation. Longitudinal tea-consumption analyses in Chinese and British cohorts found associations with clinical aging measures, although some change-to-change comparisons were statistically inconclusive [102]. These findings motivate studies of habitual intake without establishing concentrated extracts as treatments for liver aging. Figure 3 integrates the nutritional pathways and their different levels of evidence.

## 7. Dietary Interventions Improve Hepatic and Cardiometabolic Outcomes

### 7.1. Sustained Weight Reduction Links Nutritional Care to Tissue Outcomes

Sustained weight reduction is the most established nutritional target when excess adiposity is present. In a prospective study of 293 patients with biopsy-proven NASH, greater lifestyle-associated weight loss tracked histological improvement [40]. Among participants losing at least 10%, 90% had NASH resolution and 45% had fibrosis regression. These subgroup results establish neither the expected response of everyone entering treatment nor a randomized comparison. Their clinical importance lies in the graded relationship between sustained weight change and liver histology. At population level, the relevant implementation goal is to increase the proportion of eligible patients who can achieve and maintain meaningful benefit.

Treatment delivery influences the benefit that nutritional prescriptions can achieve. A seven-center Chinese trial randomized 226 adults to an intensive diet, exercise, and counseling program or a less intensive comparator [45]. Among 167 completers, the intensive strategy improved liver fat attenuation and metabolic measures without a clear additional liver-stiffness benefit. Diet composition and counseling intensity changed together, supporting evaluation of the complete care program. Attrition and the per-protocol primary analysis limit estimates of effectiveness across all enrolled participants. Reach, retention, affordability, and continuity of support should therefore accompany clinical outcomes in evaluations intended to guide public health services.

### 7.2. Mediterranean-Style Patterns Address Hepatic and Cardiovascular Risk

Mediterranean-style patterns provide a practical connection between liver-directed nutrition and broader cardiovascular prevention. DIRECT PLUS randomized 294 adults with abdominal obesity or dyslipidemia to healthy dietary guidance, a Mediterranean diet, or a green Mediterranean diet [41]. The intervention lasted 18 months and included physical activity in all groups. The green Mediterranean group achieved a larger proportional intrahepatic-fat reduction than the standard Mediterranean group (38.9% versus 19.6%), despite similar moderate weight loss. Reduced red and processed meat, walnuts, green tea, and a plant-based shake formed a multicomponent intervention, preventing attribution to a single polyphenol. The predominantly male sample and incomplete magnetic resonance follow-up constrain generalization.

Comparative trials support flexibility in achieving these nutritional goals. In MEDINA, 42 adults with NAFLD were randomized to an ad libitum Mediterranean or low-fat diet for 12 weeks [42]. The Mediterranean diet was not superior for hepatic or metabolic outcomes, and within-group improvement in the low-fat arm does not establish between-group superiority. A 250-participant MASLD trial likewise found similar CAP improvement with moderately hypocaloric Mediterranean and low-fat diets and no significant diet-by-PNPLA3 interaction [43]. Its short-term liver-stiffness changes do not establish biopsy-confirmed fibrosis regression. Together, these findings favor offering feasible alternatives and evaluating adherence and response, rather than assuming universal superiority of one named diet.

Long-term observational evidence extends beyond steatosis. Among 47,429 UK Biobank participants with MASLD followed for a median of 13.3 years, higher Mediterranean diet adherence was associated with fewer liver-related events and lower mortality [19]. Comparing high with low adherence, the reported hazard ratios were 0.553 for liver-related events and 0.854 for mortality. Associated fatty-acid and lipoprotein signatures offer plausible biological links, but observational mediation does not prove that the measured metabolites carry the dietary effect. Although alcohol contributed to the dietary score, alcohol should not be introduced as a therapeutic component of a Mediterranean-style diet for MASLD.

Cardiovascular trials strengthen the rationale for selecting patterns with benefits beyond the liver. The corrected and republished PREDIMED analysis found fewer major cardiovascular events with Mediterranean patterns supplemented by olive oil or nuts in high-risk adults [6]. Its revised analysis accounted for identified allocation deviations. CORDIOPREV found fewer major cardiovascular events with a Mediterranean than a low-fat diet in secondary prevention over seven years [7]. Although these populations were not selected to test MASLD treatment, their findings support integrating liver and cardiovascular prevention within nutritional care. This shared clinical rationale is more immediately actionable than a claim of hepatic rejuvenation.

Translation requires dietary principles that can be sustained within local food budgets and culinary traditions. Feasible choices should preserve nutrient quality while accommodating food availability, cooking capacity, preferences, and protein requirements. The trial-specific green Mediterranean regimen should not be treated as a prerequisite for effective nutritional care. Pragmatic studies should compare locally adapted patterns and document food costs, adherence, and outcomes over time. Table 4 distinguishes the direct nutritional studies from the clinical claims they can support.

### 7.3. Meal Timing Offers Flexible Routes to Energy Restriction

Time-restricted eating is an organizational option whose value depends on feasibility and the achieved energy deficit. TREATY-FLD compared an early eight-hour eating window with habitual meal timing in 88 adults with obesity and NAFLD under matched calorie prescriptions [44]. Liver fat fell in both groups over the 12 months. The between-group difference at 12 months was 1.0 percentage point (95% confidence interval, −1.6 to 3.5). A separate 139-participant obesity trial found no significant additional weight-loss advantage from time restriction when both groups received calorie restriction [103]. Meal timing can therefore be individualized to daily routines, treatment requirements, and sustained adherence, without presuming an independent effect on hepatic aging.

Diurnal metabolism remains relevant to both intervention design and measurement. A 2026 human tracer study found greater nighttime insulin resistance, DNL, and fatty-acid exposure in MASLD; aspects of the disturbance persisted after weight loss [104]. The mechanism merits targeted trials, but does not identify an optimal clinical eating window. Standardized sampling times are also necessary because diurnal variation can create apparent molecular differences unrelated to durable changes in aging.

Ketogenic diets demonstrate the importance of evaluating metabolic changes alongside steatosis. An earlier isocaloric carbohydrate-restriction study linked rapid steatosis improvement to reduced DNL, increased ketogenesis, and changes in microbial and one-carbon pathways [20]. In a 2026 crossover study, ketogenic and nonketogenic energy restriction produced similar energy deficits [21]. The ketogenic diet reduced intrahepatic triglycerides more while increasing mitochondrial redox state and suppressing tricarboxylic-acid-cycle oxidation. Those metabolic findings do not establish clinical harm, but a rapid steatosis response alone cannot establish healthier hepatic aging. Longer-term evaluation should include dietary adequacy, tolerability, cardiometabolic outcomes, and muscle function.

### 7.4. Nutrition Changes Selected Aging Measures in Populations Outside MASLD

Nutrition trials with aging endpoints establish responsiveness of selected biomarkers, predominantly in populations not selected for MASLD. They complement the hepatic intervention evidence but leave the link between dietary treatment, liver-specific aging, and clinical benefit unresolved. In CALERIE, the parent trial allocated 220 adults without obesity to a two-year calorie-restriction intervention or ad libitum intake [46]. The methylation subset showed a small slowing of DunedinPACE without significant effects on PhenoAge or GrimAge. The parent population and the assayed subset must remain distinct when interpreting the strength and applicability of this result.

Companion CALERIE analyses show that estimated effects depend on the biomarker and observation window. A 185-participant analysis found the largest effects on clinical metabolic and cardiovascular ages [47]. A modest clinical liver-age difference emerged only at 24 months (−0.54 years), without direct hepatic epigenetic or proteomic sampling. Telomere analyses found differing patterns during weight loss and maintenance, with no overall treatment difference across the full two years [105]. These results favor prespecified measurements tied to an explicit biological construct, with established metabolic outcomes retained as independent measures of benefit.

The DO-HEALTH methylation substudy assessed 777 older adults over three years [48]. Omega-3 supplementation produced modest changes in selected clocks, with reported effects corresponding to approximately 2.9–3.8 months. Vitamin D and exercise provided additive effects for PhenoAge in combination. These findings demonstrate responsiveness in an older general population; their relevance to hepatic aging or fibrosis remains to be evaluated in MASLD.

Differences across participants and measures further limit a universal dietary aging claim. In 219 postmenopausal women from the DAMA trial, dietary intervention slowed GrimAge over two years, whereas physical activity affected stochastic epigenetic mutations [49]. The NU-AGE pilot in 120 older adults showed heterogeneous changes in Horvath age [50]. The clearest signal occurred in Polish women and participants who were epigenetically older at baseline. Biological heterogeneity, subgroup multiplicity, and regression toward the mean all require consideration when interpreting that pattern.

Clinical composite ages can capture treatment-related improvements while retaining the limitations of their constituent measurements. Fasting-mimicking diet analyses reported lower hepatic fat and a 2.5-year median reduction in clinical biological age after three cycles [51]. These secondary and exploratory analyses used neither a DNAm nor a liver-specific clock. In older adults with obesity, a separate randomized trial found improvements in clinical biological-age measures with diet alone and diet plus exercise [106]. Combined treatment benefited a broader healthy-aging index, reinforcing the value of evaluating functional and metabolic outcomes together.

The MACRO trial shows why baseline risk association and treatment responsiveness require separate evaluation. Among 144 participants with obesity receiving 12 months of low-carbohydrate or low-fat dietary treatment, baseline DunedinPACE was related to several cardiometabolic markers [52]. Changes in epigenetic measures were not significantly associated with biomarker changes and did not mediate weight-loss effects. The broader review of antiaging diets in Science likewise concludes that overlapping experimental mechanisms do not resolve efficacy, tolerability, or applicability in humans [107]. The practical research objective is to identify which measurements add useful information about durable benefit after nutritional treatment. Table 5 summarizes the resulting hierarchy of evidence.

### 7.5. Observational Associations Inform Prevention Priorities and Expose Confounding

Observational studies broaden the evidence to habitual dietary patterns and help identify priorities for intervention research. In Framingham Offspring, higher Dietary Approaches to Stop Hypertension (DASH) scores were associated with slower epigenetic aging, with stronger relationships in ever-smokers [108]. In Moli-sani, Mediterranean and DASH patterns were associated with lower clinical biological age, whereas the studied Nordic and Palaeolithic scores were not [109]. These differences underscore the need to evaluate the foods captured by each score and its relevance across cultural settings.

In UK Biobank, EAT-Lancet diet adherence was associated with younger clinical biological-age estimates, with part of the association statistically attributable to adiposity [110]. The result supports examining habitual dietary patterns alongside metabolic outcomes, with careful analysis of the contribution of adiposity. NHANES analyses linked inflammatory and oxidative dietary scores to clinical age measures, although shared biomarker content and cross-sectional design limit mechanistic inference [111]. A separate NHANES study related healthier eating to lower NAFLD prevalence and examined biological-age mediation [112]. Its cross-sectional design cannot establish whether diet precedes aging changes or disease influences eating behavior.

Shared genetic liability and clustered behaviors contribute to apparent diet–aging relationships. In FinnTwin12, associations between unfavorable dietary patterns and faster epigenetic aging weakened after accounting for body mass index and physical activity [113]. Associations were generally smaller within monozygotic pairs, further supporting a role for shared determinants. Public health research should therefore assess dietary change within its behavioral and socioeconomic context. Programs can evaluate accessible food substitutions, sustained adherence, metabolic health, and physical function while retaining aging measures as complementary research outcomes.

## 8. Molecular Responses Gain Value When They Improve Clinical Decisions

### 8.1. Risk Prediction, Treatment Selection, and Monitoring Are Different Tasks

An aging measure can be useful in three distinct ways. A prognostic marker estimates future risk irrespective of treatment. A predictive marker identifies a difference in the relative benefit of alternative treatments. A response marker changes after intervention. Evidence for one role does not establish the others. In particular, an association between baseline age acceleration and poor outcome does not show which diet should be prescribed, and a change after treatment does not establish mediation of clinical benefit.

The 2026 SLIM LIVER analysis makes this distinction concrete. Among 41 people with HIV and MASLD receiving semaglutide for 24 weeks in a single-arm study, median DunedinPACE increased by 0.018 and principal-component GrimAge increased by 0.54 years [53]. Participants whose DunedinPACE decreased had greater liver-fat reduction, but that post hoc subgroup association did not demonstrate overall epigenetic rejuvenation, prospective prediction of response, or a treatment effect against a concurrent control. HIV, antiretroviral therapy, and the small sample also limit transportability. The study is useful precisely because it shows that clinical and molecular responses need not move together.

Pharmacological trials provide a clinical evidentiary benchmark rather than evidence for nutritional slowing of aging. MAESTRO-NASH evaluated resmetirom using steatohepatitis and fibrosis endpoints [114]; ESSENCE reported histological benefit with semaglutide [115]; and SYNERGY-NASH assessed MASH resolution without fibrosis worsening with tirzepatide [116]. Their relevance here is the alignment between the treatment claim, comparator, and liver outcome. An aging endpoint is not a prerequisite for clinical benefit, and clock-guided nutritional care would need to add value to established management.

**Figure 4:**
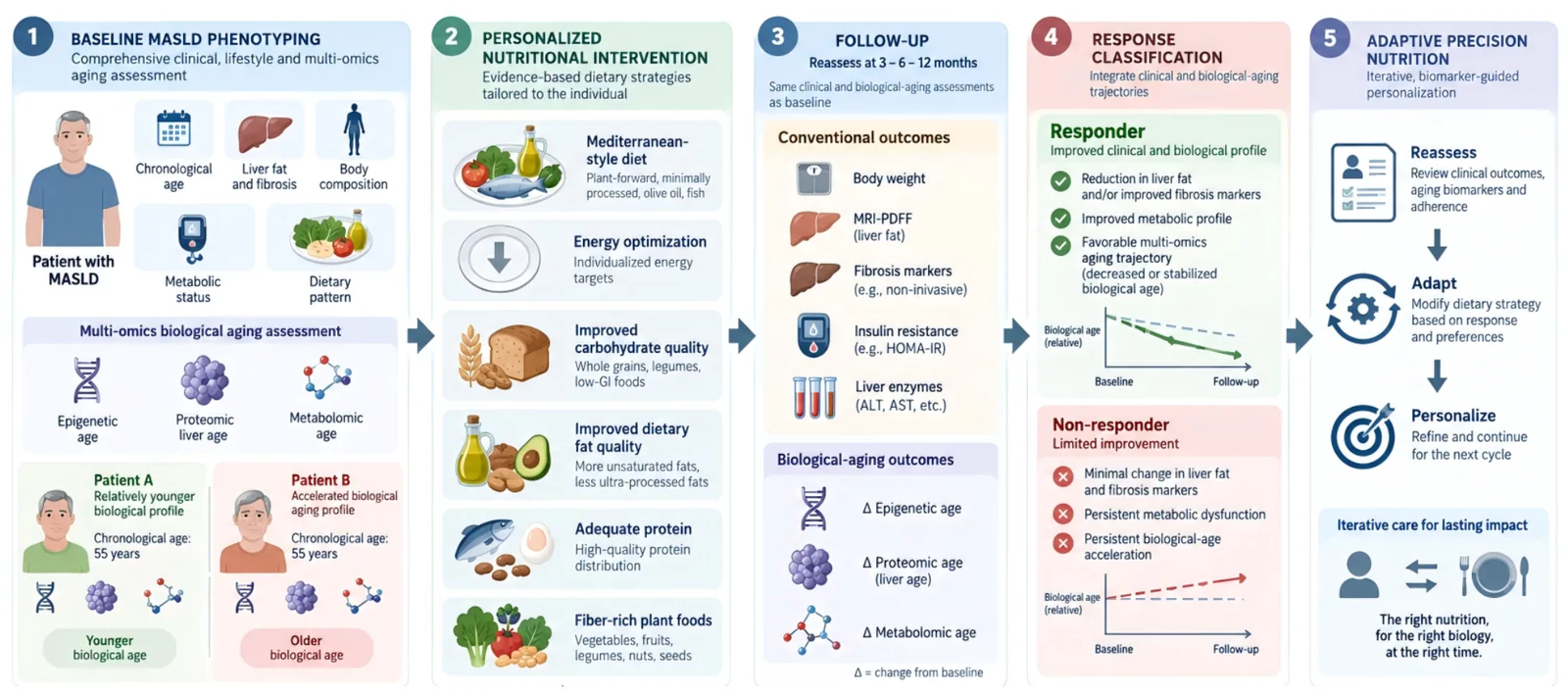
Proposed joint assessment of nutritional and biological-aging responses. The workflow is conceptual. Patient examples and plotted trajectories are illustrative, not observed data. Changes in liver fat, metabolic health, function, and aging measures should be analyzed separately before any combined response classification is considered. A falling clock value alone does not establish treatment success, and an unchanged value does not establish clinical nonresponse. The 3-, 6-, and 12-month assessments are proposed research visits, not an evidence-based clinical clock-testing schedule. MRI-PDFF, magnetic resonance imaging proton density fat fraction; GI, glycemic index; Δ, change from baseline.

Figure 4 presents an illustrative research workflow. The depicted 3-, 6-, and 12-month visits are not recommended clinical testing intervals. Its “responder” and “non-responder” labels should be interpreted as hypothetical profiles whose clinical and molecular components require separate evaluation. A patient may improve liver fat and cardiometabolic health with no detectable clock change; a favorable clock shift may coexist with worsening strength or fibrosis assessment. Reporting concordant and discordant response profiles preserves this information and avoids a clock-defined treatment decision.

### 8.2. Actionable Clinical and Dietary Phenotypes Anchor Personalization

The most useful precision nutrition variables are often available before molecular testing: food intake, energy balance, diabetes, dyslipidemia, fibrosis risk, muscle function, medication use, and the ability to obtain and prepare appropriate foods. A pathophysiology-based review of cardiometabolic phenotypes argues for tailoring nutritional strategies to tissue-level metabolic disturbances [117]. This framework is compatible with aging biology, but does not depend on having a numerical age estimate. Historical NAFLD intervention studies are used here to support principles such as sustained weight reduction, fiber adequacy, and dietary quality; because many did not report the cardiometabolic criteria required for MASLD, their transportability to contemporary MASLD is graded rather than assumed.

PREDICT 1 showed substantial interindividual variability in glycemic, lipemic, and insulin responses to identical meals and developed models that partly predicted these responses [118]. Its accompanying microbiome analysis connected habitual diet, microbial composition, and cardiometabolic profiles in 1098 deeply phenotyped participants [119]. These studies establish that meal responses differ between people and that molecular features carry information. They do not establish that microbiome-guided dietary advice improves fibrosis or that an aging clock improves the recommendation beyond simpler measurements.

MASLD-specific work is beginning to address treatment matching. In the Fatty Liver in Obesity study, inflammatory markers changed during a two-year dietary intervention, and an exploratory baseline score was used to identify which dietary strategy might yield greater weight loss [120]. This is closer to treatment prediction than a cross-sectional diet–age association, but the derived decision rule requires external validation before routine use. A Korean cohort reported a diet-by-GCKR association with MASLD prevalence [121]; that observational interaction must be distinguished from evidence of differential benefit after randomized treatment.

Troisi and colleagues’ 2026 prospective study followed 148 adults with MASLD starting a personalized hypocaloric Mediterranean diet and found weight-dependent, genotype-associated metabolomic remodeling [24]. The patterns suggest possible adherence or response biomarkers, but high classification accuracy within the study does not establish externally validated prediction of future response. The absence of a significant PNPLA3-by-diet interaction in the larger randomized Mediterranean-versus-low-fat trial remains an important counterweight [43]. Genetic susceptibility should not be translated into a prescriptive diet rule without evidence that it changes the comparative treatment effect.

**Figure 5:**
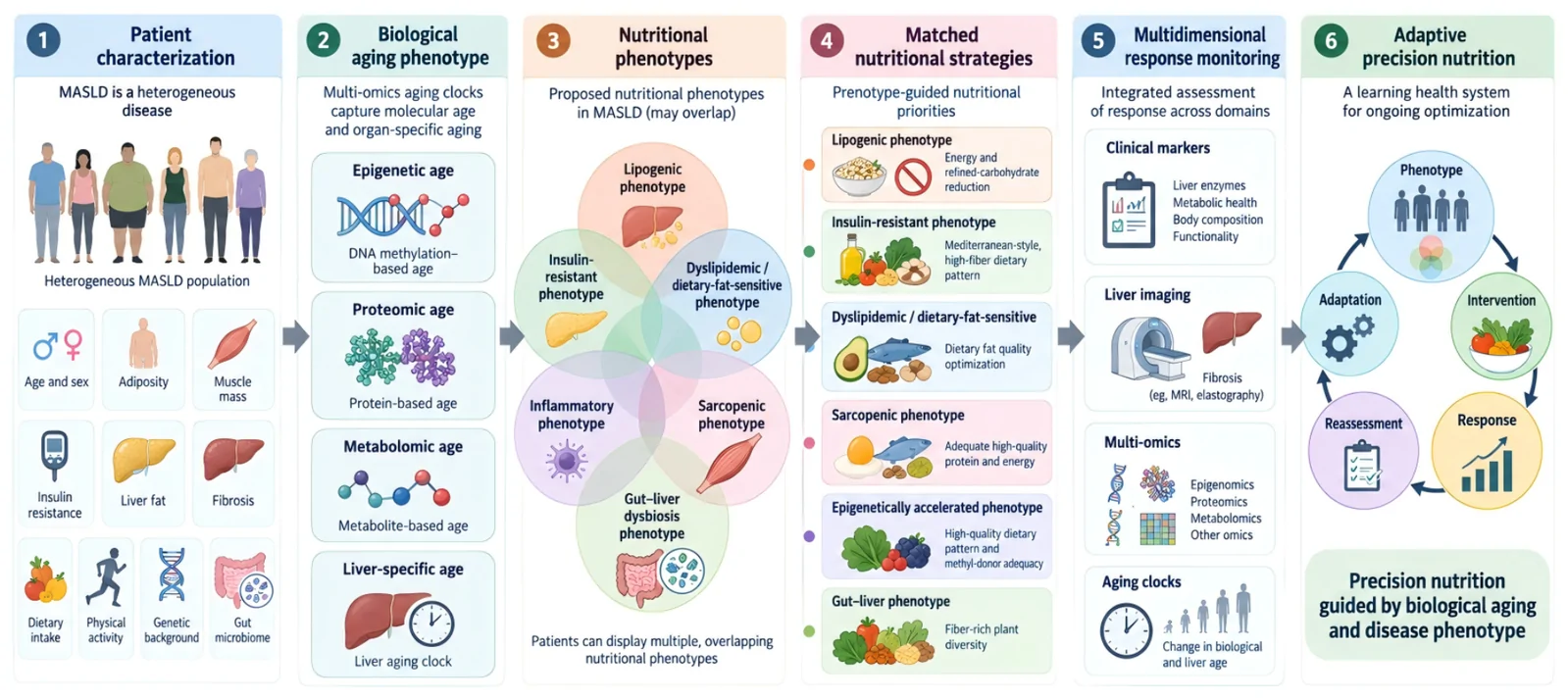
A proposed precision nutrition framework for MASLD. Clinical and dietary characterization precede any optional research assessment of biological aging. The nutritional phenotypes shown overlap and are hypotheses for testing rather than validated molecular subtypes. Dietary priorities should reflect fibrosis stage, metabolic disease, muscle reserve, preferences, and feasibility. Neither “epigenetically accelerated” nor “gut–liver dysbiosis” currently defines a validated clock-based dietary prescription; methyl-donor adequacy denotes avoidance or correction of deficiency, not empiric high-dose supplementation. Adaptive care remains anchored to clinically meaningful outcomes. MRI, magnetic resonance imaging.

Table 6 sets out a clinically anchored framework. Overlapping phenotypes identify nutritional priorities rather than mutually exclusive disease subtypes. An elevated aging score may support research enrollment, but supplementation, restrictive eating, and surveillance decisions require established clinical indications. Figure 5 illustrates a future research framework for aging-informed precision nutrition: the molecular-aging layer is optional and investigational, while clinical phenotyping supplies the current basis for care. Evidence of added treatment-selection value is needed before these layers can be given equal clinical weight.

### 8.3. Clinical Outcomes and Functional Reserve Determine Treatment Priorities

For patients with excess adiposity and noncirrhotic MASLD, follow-up should integrate weight and waist change, dietary adherence, glycemic control, lipids, blood pressure, liver enzymes, and an appropriate fibrosis-assessment pathway [4,5]. Magnetic resonance imaging proton density fat fraction (MRI-PDFF) is valuable in trials and selected clinical settings, but falling liver fat does not independently prove fibrosis regression. Similarly, short-term decreases in liver stiffness can reflect changes in inflammation as well as fibrosis. The reason for measuring each endpoint should be explicit.

Fibrosis-4 (FIB-4) includes chronological age in its formula. Its interpretation in older adults therefore differs from that in younger people; guidance recommends age-appropriate thresholds and further assessment when indicated [5]. A biological-age score should not replace the chronological-age term in FIB-4 or create a new threshold without validation. If an aging model itself contains liver enzymes or stiffness, correlations with FIB-4 or elastography should also be examined for shared components rather than described as independent confirmation.

In cirrhosis or significant frailty, preservation of intake, muscle mass, and physical function becomes central, and prolonged fasting or aggressive energy restriction may conflict with care priorities [97]. Nutrition plans should accommodate renal disease, diabetes treatment, gastrointestinal symptoms, and medication-associated appetite reduction. A liver-aging framework has clinical value only if it helps resolve these competing needs. In many settings, access to a dietitian, food affordability, and continuity of follow-up will matter more than adding an expensive molecular assay.

## 9. Public Health Implementation Connects Dietary Prevention to Risk-Based Care

### 9.1. Population Prevention and Clinical Care Address Complementary Needs

The clinical evidence supports an implementation agenda extending beyond individualized dietary prescriptions. The global public health consensus for NAFLD connects liver disease with wider noncommunicable disease policies and coordinated health-system responses [122]. At population level, prevention addresses cumulative nutritional exposures before advanced disease develops. At clinical level, nutritional care must respond to existing fibrosis risk, cardiometabolic disease, and functional vulnerability. These approaches share practical dietary priorities, including replacing sugar-sweetened beverages, improving fat quality, increasing tolerated fiber-rich foods, and maintaining adequate protein [4,5]. Their intensity and delivery should reflect need rather than access to molecular testing.

Food affordability, availability, and preparation demands influence the feasibility of sustained change. Public health programs should therefore pair dietary advice with assessment of practical barriers and locally appropriate food options. A Mediterranean-style pattern provides a useful nutritional model, but implementation requires culturally acceptable substitutions rather than a fixed list of specialty foods. Evidence of dietary efficacy does not establish the effectiveness of any particular food subsidy, procurement policy, or service model. Those strategies require evaluation of uptake, sustained dietary change, and health outcomes in the populations they serve.

### 9.2. Primary Care Integration Makes Prevention Clinically Actionable

MASLD care can be linked to existing pathways for obesity, type 2 diabetes, and cardiovascular risk. A synthesis of NAFLD care models emphasizes coordination of services, settings, professional roles, and referral pathways [123]. Current guidance supports case finding in people at increased risk, followed by sequential noninvasive fibrosis assessment when indicated [4,5]. This differs from universal screening of asymptomatic populations, and neither pathway requires an aging-clock assay. Dietary and functional assessment can accompany routine metabolic follow-up, with specialist referral determined by established clinical criteria. Public health value depends on completing this pathway, including access to counseling and continued support after risk identification.

Older adults require particular attention because weight loss and preserved function can become competing objectives. Programs should assess intake, strength, and frailty alongside metabolic response, especially in cirrhosis [97]. This life-course perspective connects earlier reduction of cumulative metabolic burden to later preservation of independence. It does not assume that the same energy restriction or meal schedule is appropriate at every age or disease stage. Table 7 translates these principles into proposed implementation priorities and evaluation measures.

### 9.3. Metabolic Biomarkers Must Demonstrate Value Under Real Service Conditions

Metabolomic profiling may help distinguish dietary exposure, adherence, and metabolic response when routine measurements leave an actionable uncertainty. Its value depends on reproducibility, interpretable pathways, and evidence that the information changes a useful decision. A responsive fatty-acid or microbial-metabolite signature may support exposure assessment without functioning as an aging biomarker [18,22]. Population deployment additionally requires feasible collection, analytical standardization, acceptable costs, and performance across diverse dietary and clinical settings.

Implementation studies should report reach, retention, nutritional adequacy, functional outcomes, and resource use alongside hepatic and cardiometabolic endpoints. Uptake and benefit should be examined across socioeconomic circumstances, geography, sex, age, and relevant clinical subgroups. These are proposed evaluation requirements, not demonstrated effects of clock-guided care. The policy objective is to expand effective nutritional support while establishing whether additional molecular information yields sufficient benefit to justify its cost.

## 10. Biomarker Qualification Connects Metabolic Information to Clinical Utility

The evidence gap is a missing link between nutritional efficacy and biomarker utility. Trials such as DIRECT PLUS and TREATY-FLD evaluate dietary effects on liver fat; CALERIE and DO-HEALTH evaluate aging measures in different populations; MASLD tissue and prognostic studies establish disease associations. SLIM LIVER links MASLD treatment and systemic clocks, but uses a drug intervention and a single-arm design. None supplies the complete nutrition–liver-specific aging–clinical benefit chain. Table 8 specifies the evidence needed to connect these strands. The sequence is a qualification framework rather than a claim that every useful prognostic marker must become a surrogate.

### 10.1. Prospective Comparison Establishes the Added Value of Biomarker-Guided Care

Clinical utility testing requires a reproducible assay and a prespecified decision rule supported by prior validation. Once these conditions are met, a trial can compare clinically individualized nutrition with the same program augmented by the locked biomarker-guided algorithm. Both groups should receive equivalent counseling intensity and access to food support. Otherwise, better outcomes could reflect attention or resources rather than the biomarker. Earlier mechanistic feeding studies can compare dietary composition under closely matched energy intake and test temporal relationships between metabolic responses and aging measures.

Enrollment should document the MASLD definition, baseline fibrosis assessment, diabetes status, medications, sex, age, and muscle function. Noncirrhotic and cirrhotic populations should be studied separately when nutritional goals differ. Prespecified strata may test heterogeneity, but a continuous baseline clock measure is generally more informative than an arbitrary “old” versus “young” cutoff. Sample size must be based on the chosen clinical endpoint and its expected variability; a treatment-by-biomarker interaction usually requires more information than a main treatment effect.

### 10.2. Clinical Efficacy and Biomarker Qualification Require Different Endpoints

The primary endpoint should match the proposed use. A short efficacy trial might prioritize a prespecified change in MRI-PDFF with metabolic and functional safety outcomes. An antifibrotic claim requires a suitable validated fibrosis outcome and adequate follow-up. Longer studies should assess liver-related events, cardiometabolic events, physical function, and quality of life. Molecular clocks should initially be secondary or exploratory outcomes, with a limited prespecified panel and appropriate control of multiplicity.

An early metabolomic assessment may identify exposure and adherence, while repeated epigenetic and proteomic measurements over longer intervals can test persistence. Baseline replicate samples in a subset, balanced laboratory batches, blinded assays, fixed preprocessing, and a locked model are necessary to interpret changes of the magnitude seen in nutrition trials [39,88]. Sample timing, fasting, acute illness, medication changes, and renal function should be documented. Time of day deserves explicit control given the human diurnal findings in MASLD [104].

Clock-guided treatment requires prospective evidence of a treatment-by-biomarker interaction, followed by validation of the resulting decision rule in an independent population. Prognostic performance should be judged against strong clinical comparators using calibration and decision-relevant benefit as well as discrimination. Economic evaluation should ask whether testing improves outcomes sufficiently to justify its cost and whether it widens disparities in access to nutritional care.

### 10.3. Temporal Evidence and Discordant Responses Clarify Biological Interpretation

Weight loss can be an intended mechanism of dietary benefit. Adjusting for it in every model can remove part of the total effect that the trial is designed to estimate. Analyses should therefore distinguish the total treatment effect from a prespecified effect not operating through weight change. Mediation involving an aging clock additionally requires temporal ordering, control of mediator–outcome confounding, and consideration of measurement error; simultaneous changes measured at one follow-up visit are insufficient to establish a causal sequence.

The decisive validation is whether treatment-induced clock changes reliably predict later clinical benefit across interventions and populations. A statistically significant association within one trial is an initial observation, not surrogate-endpoint validation. The discordant findings from MACRO and SLIM LIVER make this an empirical question rather than an assumed property of aging clocks [52,53]. Until it is resolved, reporting parallel clinical, functional, and molecular outcomes offers a more useful picture than combining them into a single responder label.

## 11. Evidence Directness and Transportability Bound the Interpretation

The main strength of this evidence base is triangulation: randomized nutritional trials establish effects on selected hepatic or systemic outcomes, tissue studies connect aging measures to pathology, and experimental work identifies plausible mechanisms. Their convergence supports a biological rationale for investigation. The main limitation is directness. Small liver-biopsy studies and MASLD association cohorts do not establish dietary responsiveness, while most nutrition trials with aging endpoints sample blood in populations not selected for MASLD. Historical NAFLD eligibility, differing fibrosis distributions, clock training targets, sampling intervals, and assay platforms further limit comparisons. A change in a clinical composite may also partly reproduce changes in its constituent liver or metabolic measurements.

Transportability requires explicit testing across ancestry, sex, and age. European-ancestry discovery cohorts, selected biobank volunteers, predominantly male dietary trials, and older-adult intervention studies cover different combinations of exposure and vulnerability. External age prediction is encouraging, but clinical calibration and treatment-response performance can still differ with baseline disease, body composition, medications, diet, and laboratory platform. Sex differences in experimental dietary responses and the heterogeneous NU-AGE findings illustrate why a pooled average may not define an individual prescription [50,81]. Validation should examine clinically relevant subgroups and calibration without interpreting underpowered subgroup contrasts as established biological differences.

This review uses purposeful narrative selection and qualitative appraisal. Although it includes discordant and null findings, selection and publication bias remain possible, and it does not estimate their magnitude or provide a pooled treatment effect. Studies with many clocks, time points, or exploratory subgroups are especially vulnerable to selective emphasis. Observational diet associations remain affected by socioeconomic factors, adiposity, reverse causation, and medication exposure; cross-sectional mediation adds no temporal ordering. These limitations define the next research need: prospective MASLD nutrition studies with prespecified aging measures, standardized sampling, functional safety outcomes, and independent validation against established clinical assessment.

## 12. Conclusions

Nutrition can improve selected hepatic and cardiometabolic outcomes relevant to MASLD and provides a practical foundation for prevention and sustained care. Its public health value lies in addressing cumulative dietary risk, supporting metabolic health, and preserving function through accessible services. Fibrosis risk, muscle reserve, comorbidity, and food access determine how these priorities should be implemented. Direct evidence that nutrition slows liver-specific biological aging remains absent: the required combination of a defined MASLD population, a nutritional intervention, and a liver-tissue or independently validated liver-specific aging endpoint has not yet been demonstrated.

Metabolic remodeling provides a biological framework for nutritional benefit, and metabolomics offers tools to characterize exposure and response. Epigenetic and proteomic aging measures can complement that information, but each proposed use requires independent qualification as an exposure marker, association biomarker, prognostic biomarker, response biomarker, or candidate surrogate endpoint. Reduced steatosis, altered metabolites, a lower composite score, and fibrosis regression remain distinct outcomes. Aging-informed precision nutrition will advance care only when molecular information improves decisions, durable health outcomes, and equitable delivery beyond established clinical assessment.

## Figures and Tables

**Table 1:** Selected human evidence linking biological aging to MASLD and clinically relevant outcomes, with original disease definitions and transportability.

Study	Original Disease Definition	Population and Design	Main Observation	Validated Role	MASLD Transportability
Horvath et al., 2014 [10]	Obesity and NAFLD-spectrum liver tissue; historical histology, not MASLD criteria.	Human tissues; two liver datasets; observational and short-term post-bariatric observations.	Hepatic DNAm age increased by approximately 3.3 years per 10 BMI units; no rapid reversal after surgical weight loss.	Association biomarker; tissue specificity, not a treatment-response or surrogate endpoint.	Limited/uncertain: not selected for MASLD and driven by BMI; useful for biology rather than case definition.
Loomba et al., 2018 [11]	Biopsy-proven NASH with F2–F3 fibrosis under historical NAFLD criteria.	Peripheral-blood methylation with comparison cohorts.	Age acceleration correlated with quantitative collagen content, but did not distinguish F2 from F3.	Association biomarker; fibrosis-linked signal, not a stage classifier.	Moderate to high for advanced MASLD/MASH with fibrosis; alcohol exclusion and cardiometabolic criteria were not identical.
Xia et al., 2024 [12]	NAFLD by ultrasound/clinical criteria; pre-MASLD terminology.	Shanghai Changfeng cohort; blood methylation with approximately four-year liver-fat follow-up.	Highest versus lowest acceleration tertile: adjusted NAFLD odds ratio 4.55 (95% CI 1.06–19.61); larger subsequent liver-fat increase.	Prognostic/association biomarker; not independently validated for treatment selection.	Moderate: likely cardiometabolic overlap, but alcohol exclusion and MASLD criteria were not fully aligned.
Tong et al., 2024 [13]	MASLD harmonized from NHANES variables; advanced fibrosis assessed separately.	NHANES, 3974 adults; cross-sectional clinical KDM and PhenoAge measures.	Advanced fibrosis was associated with older biological age; adjusted MASLD associations were not significant.	Association biomarker; not a prospective prognostic or response endpoint.	High for the harmonized population; cross-sectional design limits transport to treated clinical cohorts.
Fibrosis-clock comparison, 2025 [14]	MASLD defined using contemporary criteria; fibrosis grades compared.	325 individuals; five epigenetic measures compared across fibrosis grades.	Advanced fibrosis: DunedinPACE coefficient 0.05 (95% CI 0.03–0.07); no significant association with IEAA, PhenoAA, or GrimAA.	Association biomarker; clock-specific fibrosis signal, not a surrogate endpoint.	High for MASLD fibrosis research; independent ancestry, intervention, and longitudinal validation remain limited.
Van Dijck et al., 2025 [15]	MASLD spectrum diagnosed by contemporary clinical/pathological terminology.	22 liver biopsies; methylation profiling with integrative expression analysis.	Histological associations differed among Horvath, LiverClock, HepClock, and intrinsic acceleration.	Tissue association biomarker; mechanistic hypothesis generation.	Moderate to high for liver tissue; small biopsy sample and mixed cellular composition limit generalization.
Liver Aging Index, 2026 [16]	Multicohort liver disease risk definitions; not uniformly MASLD-enriched.	Development: 21,629; external cohorts: 3412 and 12,170 participants.	Mortality discrimination approximately 0.76 in validation cohorts; associations with liver-related events.	Candidate prognostic biomarker; treatment-response and surrogate roles unvalidated.	Moderate: liver-risk transportability is plausible, but MASLD-specific calibration and treatment response require testing.
MASLD-related HCC study, 2026 [17]	MASLD-related HCC cases and cancer-free MASLD controls under contemporary terminology.	Case–control association; 272 cases and 316 controls across six sites.	PCPhenoAge, PCGrimAge, and DunedinPACE associated with case status; PCHorvathAge and PCHannumAge did not.	Association biomarker; case discrimination is not prospective cancer prognosis.	High for the sampled MASLD-HCC spectrum; surveillance utility and prospective calibration remain unproven.

BMI, body mass index; CI, confidence interval; DNAm, DNA methylation; HCC, hepatocellular carcinoma; IEAA, intrinsic epigenetic age acceleration; PhenoAA, PhenoAge acceleration; GrimAA, GrimAge acceleration; KDM, Klemera–Doubal method; MASLD, metabolic dysfunction-associated steatotic liver disease; NAFLD, nonalcoholic fatty liver disease; NASH, nonalcoholic steatohepatitis; NHANES, National Health and Nutrition Examination Survey; PC, principal-component version. NAFLD/NASH terminology is retained when describing original study populations. Transportability judgments are qualitative and do not replace external validation.

**Table 2:** Structured synthesis of metabolomic applications relevant to nutrition, MASLD, and biological aging.

Metabolomic Application	Representative Evidence	Sample Type	Platform and Scope	Population and Nutritional Exposure	Principal Metabolite Classes	Replication/External Validation
Exposure/adherence biomarkers	Dietary fatty-acid biomarker comparison [18]; Mediterranean-associated signatures [19]	Plasma/serum	GC, NMR, and LC–MS platform comparison; targeted fatty-acid and lipoprotein panels	Adults in feeding or prospective cohort studies; dietary fatty-acid intake or Mediterranean-pattern adherence.	Fatty acids, acylcarnitines, triglyceride/lipoprotein particles, amino acids.	Cross-platform agreement is incomplete and platform dependent; adherence markers lack independent MASLD treatment validation.
Mechanistic/pathway biomarkers	Carbohydrate-restricted diet study [20]; ketogenic MASLD crossover [21]; resistant-starch trial [22]	Plasma/serum, with liver or stool measures in paired experiments	Integrated untargeted or investigational MS/NMR metabolomics; tracer-supported pathway analysis where available	Adults with obesity or MASLD receiving carbohydrate restriction, ketogenic energy restriction, or resistant starch.	Ketone bodies, branched-chain amino acids, ceramides, acylglycerols, bile acids, TCA/redox intermediates, microbial metabolites.	Mechanistic coherence and experimental triangulation are reported; no independent validation of a metabolite panel as a liver-aging surrogate.
Response biomarkers	High-fiber pilot [23]; resistant-starch response [22]; genotype-stratified Mediterranean cohort [24]	Serum/plasma; stool microbiome when paired	LC–MS/NMR metabolomics or targeted panels (assay specific); exploratory longitudinal profiling	Patients with NAFLD/MASLD or metabolic risk after fiber, hypocaloric Mediterranean, or dietary weight-loss interventions.	Short-chain fatty acids, amino acids, bile acids, lipids, microbial co-metabolites.	Mostly internal or pilot analyses; the 2026 cohort requires independent validation of response classification across diets and populations.
Prognostic signatures	Mediterranean diet metabolite signatures and MASLD outcomes [19]	Plasma	NMR metabolomics panel with multivariable signature construction	47,429 UK Biobank adults with MASLD followed for liver-related events and mortality; habitual diet exposure.	Lipoprotein subclasses, fatty acids, amino acids, ketone-related and inflammatory metabolites.	Longitudinal outcome association in one large cohort; calibration, ancestry transport, and incremental value beyond clinical risk remain to be tested.
Metabolomic aging scores	MetaboAge, MetaboAgeMort, and related aging signatures [25,26,27,28]	Plasma/serum	NMR and mass spectrometry; targeted or semi-targeted age/mortality models, study specific	General-population cohorts with habitual lifestyle or dietary exposures; not selected for MASLD intervention.	Branched-chain and aromatic amino acids, lipid and lipoprotein species, glucose-related metabolites, inflammation/redox markers.	Independent cohorts support age or mortality prediction for selected models; MASLD-specific responsiveness, treatment selection, and surrogate validity are unproven.

GC, gas chromatography; LC–MS, liquid chromatography–mass spectrometry; MASLD, metabolic dysfunction-associated steatotic liver disease; NAFLD, nonalcoholic fatty liver disease; NMR, nuclear magnetic resonance; TCA, tricarboxylic acid. Platform labels and validation statements follow the cited reports; rapid dietary shifts should not be interpreted as slower liver-specific biological aging without independent qualification.

**Table 3:** Aging measures relevant to nutritional research in MASLD: intended validation role, what they measure, and what they cannot yet establish.

Measure	Data and Construction	Potential Role	Validated Role to Date	Main Interpretive Constraint
Clinical KDM/PhenoAge [13,29]	Routine biochemical/clinical variables; chronological-age or mortality-related calibration.	Accessible epidemiology and assessment of systemic metabolic burden.	Association and prognostic biomarker in selected populations; not a validated treatment-response marker.	Not liver specific; dietary changes can directly alter score components. Clinical PhenoAge is distinct from DNAm PhenoAge.
Horvath/Hannum [30,31]	DNAm; chronological-age training; multi-tissue or blood-focused development.	Foundational comparison, tissue studies, and long-term associations.	Association biomarker; response and surrogate roles remain unvalidated.	Good age prediction does not imply sensitivity to nutritional response or specificity for fibrosis.
DNAm PhenoAge/GrimAge [29,32]	DNAm trained against health-related targets; GrimAge includes DNAm surrogates of proteins and smoking.	Risk assessment and selected intervention studies.	Association/prognostic biomarker; intervention responsiveness is measure-specific and not a surrogate endpoint.	Methylation surrogates are not measured plasma proteins; smoking and blood-cell composition affect interpretation.
DunedinPACE [14,33]	Blood DNAm trained on two decades of multisystem physiological change.	Candidate pace measure in intervention trials; direct fibrosis associations.	Association biomarker and candidate response biomarker; no validated treatment-selection threshold for MASLD.	A systemic pace estimate, not liver age in years.
Liver tissue clocks [15]	Liver DNAm; different tissue-specific or multi-tissue training approaches.	Mechanistic linkage to hepatic pathology.	Tissue association biomarker; clinical prognostic, response, and surrogate roles are unvalidated.	Biopsy sampling, cell composition, and small validation cohorts; tissue-specific labels do not ensure clinical superiority.
Global proteomic clocks [34,35]	High-dimensional plasma proteins; chronological age or mortality targets.	Multimorbidity and mortality prediction.	Prognostic biomarker in broad cohorts; MASLD response utility not established.	Platform and population dependence; incremental clinical benefit can be modest despite strong associations.
Organ-specific proteomic clocks [36,37,38]	Organ-enriched proteins measured in plasma; age, mortality, or longitudinal training.	Organ-level heterogeneity and hypothesis generation.	Candidate association/prognostic biomarker; response and surrogate roles require external testing.	Organ attribution is indirect; clearance, secretion, and injury affect circulating abundance. Nutritional response needs validation.
MetaboAge, MetaboHealth, MetaboAgeMort [26,27]	NMR or mass-spectrometry metabolites; age- or mortality-trained models.	Exposure-sensitive metabolic phenotyping and candidate monitoring.	Candidate prognostic/aging biomarker; nutritional response and MASLD treatment-selection roles are unvalidated.	Acute diet, fasting, ketosis, and renal function can move a score independently of tissue aging. Models are not interchangeable.
Liver Aging Index [16]	Clinical and plasma biomarkers plus fat attenuation and liver stiffness; mortality-calibrated construction.	Candidate clinical prognostic assessment where assays and imaging are available.	Prognostic biomarker with external cohort validation; treatment-response and surrogate roles remain unproven.	Shares inputs with conventional liver/metabolic outcomes; independent treatment-response utility remains unproven.
Integrated multi-omics approach [39]	Prespecified combination of methylation, proteins, metabolites, and clinical phenotype.	Research framework for complementary signals and discordant response patterns.	Investigational framework; no validated composite response or surrogate endpoint.	No single validated integrated score currently justifies clock-guided nutritional prescriptions in MASLD.

DNAm, DNA methylation; KDM, Klemera–Doubal method; NMR, nuclear magnetic resonance. Association, prognostic, exposure, response, predictive, and surrogate uses require distinct validation and should not be inferred from one another.

**Table 4:** Nutritional intervention evidence for hepatic outcomes and its relevance to liver-aging research, with original disease definitions and transportability.

Study	Original Disease Definition	Design and Exposure	Hepatic/Molecular Result	Biomarker Validation Role	MASLD Transportability
Lifestyle weight loss [40]	Biopsy-proven NASH under historical NAFLD criteria.	Prospective cohort; 293 with NASH; 52 weeks; 261 paired biopsies.	In the subgroup losing ≥10% weight: 90% NASH resolution and 45% fibrosis regression.	Clinical efficacy evidence; no aging-biomarker or surrogate validation.	Moderate to high for substantial fibrosis-risk MASLD; subgroup and historical eligibility limit generalization.
DIRECT PLUS [41]	Abdominal obesity/dyslipidemia; not a MASLD-defined cohort.	RCT; 294 adults; 18 months; green Mediterranean, Mediterranean, or healthy guidance, all with activity.	Relative intrahepatic-fat changes: −38.9%, −19.6%, and −12.2%, respectively.	Clinical response evidence; no validated molecular response or aging endpoint.	Limited to moderate: cardiometabolic risk is relevant, but disease status and fibrosis spectrum were not uniformly defined.
MEDINA [42]	NAFLD by historical clinical criteria.	RCT; 42 with NAFLD; 12 weeks; ad libitum Mediterranean versus low-fat diet.	No significant between-group difference in hepatic or metabolic outcomes.	Clinical comparative efficacy; negative result does not validate a biomarker.	Moderate: likely overlap with MASLD, but alcohol and cardiometabolic criteria were not aligned.
Mediterranean versus low-fat, 2025 [43]	MASLD defined using contemporary terminology.	RCT; 250 adults with MASLD; 12-week moderately hypocaloric diets.	CAP difference −0.13 dB/m (95% CI −8.54 to 8.28); no significant PNPLA3 interaction.	Clinical response evidence; genotype-by-diet prediction not validated externally.	High for short-term noncirrhotic MASLD; duration and fibrosis endpoints limit broader inference.
TREATY-FLD [44]	Obesity/NAFLD under historical criteria.	RCT; 88 adults with obesity/NAFLD; 12 months; early eating window versus habitual timing, matched calorie targets.	Liver-fat difference at 12 months: 1.0 percentage point (95% CI −1.6 to 3.5).	Clinical efficacy evidence; no aging or metabolomic surrogate validation.	Moderate: obesity and steatosis are relevant, but criteria and fibrosis distribution may differ from MASLD care.
Resistant starch [22]	NAFLD by historical diagnostic criteria.	Four-month placebo-controlled RCT; 99 intervention and 97 control participants in reported analysis.	Reported absolute intrahepatic-triglyceride reduction versus control: 9.08 percentage points; 5.89 after weight-loss adjustment.	Mechanistic response biomarker (microbiome/metabolites); no independent treatment-prediction or surrogate validation.	Moderate: dietary mechanism is relevant, but NAFLD eligibility and short follow-up limit transport.
Chinese multicenter lifestyle trial [45]	Overweight/obesity with NAFLD under historical criteria.	RCT; 226 randomized, 167 completers; 12 weeks; diet, exercise, and enhanced counseling.	Greater improvement in fat attenuation and metabolic measures; no clear additional stiffness benefit.	Clinical program response; molecular measures were not validated as treatment guides.	Moderate: intensive-care delivery is relevant, but combined components and attrition limit transport.
Ketogenic crossover study, 2026 [21]	MASLD defined using contemporary terminology.	Short-term hypocaloric ketogenic versus nonketogenic diets; tracer studies and metabolomics.	Intrahepatic triglycerides −29% versus −20%; ketogenic diet increased redox state and suppressed TCA-cycle oxidation.	Mechanistic pathway response biomarker; no durable clinical or surrogate validation.	High for short-term metabolic MASLD physiology; long-term safety, adherence, and fibrosis transport remain uncertain.
High-fiber pilot [23]	NAFLD under historical criteria; uncontrolled pilot.	27 patients; two high-fiber buns daily for two months.	CAP decreased from 309 to 277 dB/m; serum metabolites changed, including decreases in some SCFAs.	Exploratory response biomarker; internal pilot only, without comparator or external validation.	Moderate to low: small selected sample and uncontrolled design limit transport.
Genotype-stratified Mediterranean cohort, 2026 [24]	MASLD defined using contemporary terminology.	Prospective study; 148 adults starting personalized hypocaloric Mediterranean treatment.	Approximately 8% weight reduction, nearly 17% CAP reduction, and weight/genotype-associated metabolic remodeling.	Candidate exposure/response biomarkers; internal classification accuracy is not independent treatment prediction.	High for the studied MASLD setting; external validation across diets and populations is required.

RCT, randomized controlled trial; CAP, controlled attenuation parameter; CI, confidence interval; MASLD, metabolic dysfunction-associated steatotic liver disease; NAFLD, nonalcoholic fatty liver disease; NASH, nonalcoholic steatohepatitis; PNPLA3, patatin-like phospholipase domain-containing 3; SCFA, short-chain fatty acid; TCA, tricarboxylic acid. Relative percentage changes and absolute percentage-point differences are distinguished explicitly. None of these findings alone establishes slower liver-specific biological aging. Transportability judgments are qualitative and do not replace external validation.

**Table 5:** Interventions measuring biological-aging outcomes: original disease definition, biomarker validation role, directness, and implications for MASLD.

Study	Original Disease Definition	Population/Intervention	Aging Result	Biomarker Validation Role	MASLD Transportability
CALERIE DNAm analysis [46]	No liver disease required; healthy adults without obesity.	220 randomized adults; DNAm analysis in 197; two-year calorie restriction versus ad libitum control.	Small slowing of DunedinPACE; no significant treatment effect on PhenoAge or GrimAge.	Response biomarker evidence for selected clocks; no MASLD or liver-specific surrogate validation.	Limited: strong randomized intervention design but no MASLD enrichment.
CALERIE organ-age analysis, 2026 [47]	No liver disease required; healthy adults without obesity.	185 participants; clinical organ-age estimates at 12/24 months.	Stronger metabolic/cardiovascular effects; liver-age difference −0.54 years at 24 months.	Exploratory response measure; clinical composite is not a validated hepatic molecular surrogate.	Limited: organ-age estimates need MASLD calibration and independent response testing.
DO-HEALTH [48]	General older-adult population; no MASLD definition.	777 older adults in methylation substudy; three years; omega-3, vitamin D, and exercise factorial interventions.	Small omega-3 effects across selected clocks; approximately 2.9–3.8 months; additive PhenoAge effect with combined interventions.	Response biomarker evidence in a general population; no MASLD prognostic or surrogate validation.	Limited to moderate: older-adult implementation is relevant, but liver disease and fibrosis were not targeted.
DAMA [49]	Postmenopausal women; no liver disease criterion.	219 postmenopausal women; two-year dietary/physical-activity factorial trial.	Diet slowed GrimAge; activity affected stochastic epigenetic mutation burden.	Measure-specific response signals; predictive and surrogate roles unvalidated.	Limited: sex, age, and disease-selection differences constrain MASLD transport.
NU-AGE pilot [50]	Older adults without a MASLD selection criterion.	120 older adults from Italy/Poland; one-year Mediterranean-like intervention.	Heterogeneous Horvath-age response; strongest signals in Polish women and those older epigenetically at baseline.	Exploratory response biomarker; subgroup signal requires replication.	Limited to moderate: dietary pattern is relevant, but sample and disease spectrum are not MASLD representative.
Fasting-mimicking diet [51]	Adult intervention samples; no uniform MASLD definition.	Secondary/exploratory analyses of adult intervention studies; three cycles.	Median clinical biological-age reduction of 2.5 years with metabolic/immune changes and lower liver fat in analyzed samples.	Exploratory composite response measure; not DNAm or liver-specific and not a surrogate endpoint.	Limited: lower liver fat is relevant, but heterogeneous enrollment and exploratory analysis limit transport.
MACRO [52]	Adults with obesity; no MASLD requirement.	144 adults with obesity; 12-month low-carbohydrate versus low-fat weight-loss intervention.	Clock changes did not significantly track cardiometabolic changes or mediate weight-loss effects.	Negative response-validity evidence; baseline association does not establish predictive utility.	Limited to moderate: obesity is common in MASLD, but liver outcomes and disease definition were absent.
SLIM LIVER [53]	HIV-associated MASLD under contemporary terminology; pharmacological intervention.	41 people with HIV/MASLD; post hoc analysis of a 24-week single-arm semaglutide study.	Median DunedinPACE +0.018; PCGrimAge +0.54 years. Decreased pace in a subgroup associated with greater fat reduction.	Candidate response biomarker; post hoc subgroup and no randomized proof of mediation or surrogate validity.	Moderate to high for HIV-associated MASLD physiology; not dietary evidence and broader MASLD transport requires testing.

DNAm, DNA methylation; HIV, human immunodeficiency virus; MASLD, metabolic dysfunction-associated steatotic liver disease; PC, principal-component version. Sample sizes refer to the stated parent trial or biomarker analysis and should not be interchanged. These studies assess different biological constructs and are not pooled as equivalent treatment effects. Transportability judgments are qualitative.

**Table 6:** A clinically anchored precision nutrition framework: established priorities and investigational additions.

Clinical Presentation	Nutritional Priority	Monitor for Clinical Benefit	Role of Aging/Omics Measures
Excess adiposity; high sugar-sweetened beverage intake [4,94]	Feasible energy reduction; replace sweetened beverages; improve food quality with adequate protein.	Weight/waist, dietary adherence, glycemic control, liver fat when indicated, and strength.	Exposure marker (metabolites) may document intake. A lower clock value is not needed to justify effective standard care.
Insulin resistance or type 2 diabetes [5,43]	Mediterranean-style or another acceptable high-quality pattern; coordinate with glucose-lowering treatment.	Glycemia, lipids, blood pressure, liver assessment, hypoglycemia and inadequate intake.	Candidate response biomarker only if it adds information beyond baseline metabolic status; no universal best diet from one clock.
Dyslipidemia; high saturated-fat intake [18,96]	Substitute unsaturated fats within the energy target; reduce excess saturated fat.	Lipid profile, weight, adherence, and liver outcomes appropriate to baseline risk.	Exposure biomarker (fatty-acid profile) may support adherence; distinguish this role from age estimates and treatment prediction.
Low fiber or limited plant-food diversity [22,23]	Increase tolerated whole-food fiber; consider the evidence for the specific intervention and food format.	Diet quality, gastrointestinal tolerance, metabolic status, and steatosis when indicated.	Candidate mechanistic/response biomarker; microbial/metabolite changes are research adjuncts, not validated surrogate endpoints.
Sarcopenia, frailty, or cirrhosis [56,97]	Maintain adequate energy and protein; avoid prolonged fasting; integrate suitable physical activity and specialist care.	Intake, weight trajectory, muscle strength, mobility, decompensation risk, and quality of life.	Functional outcomes are primary; a molecular age estimate cannot compensate for functional decline, and clock-guided protein restriction is unsupported.
Advanced fibrosis with apparent age acceleration [4,14,16]	Stage-appropriate dietary care and indicated disease-directed management; address cardiometabolic risk.	Validated fibrosis pathways and liver-related risk; appropriate surveillance and referral.	Candidate prognostic biomarker for research risk enrichment only; do not substitute biological age into FIB-4 or use it as a surrogate endpoint.
Suspected micronutrient deficiency [99,100]	Assess and correct a demonstrated deficiency; consider renal function and other causes of abnormal biomarkers.	Nutrient status, clinical response, dietary adequacy, and relevant adverse effects.	Association biomarker (methylation/nutrient status); associations do not justify high-dose supplementation in nutritionally replete individuals.

This is a synthesis for clinical reasoning, not a validated treatment algorithm. Phenotypes overlap. Choices must accommodate comorbidities, medications, culture, affordability, preferences, and access to follow-up. SCFA, short-chain fatty acid; FIB-4, Fibrosis-4 index.

**Table 7:** Proposed public health implementation priorities for nutritional prevention and MASLD care.

Setting	Actionable Priority	Outcomes for Evaluation	Role of Metabolic and Aging Measures
Population prevention	Support affordable, culturally acceptable dietary quality; reduce sustained excess energy exposure where relevant.	Program reach, food access, dietary change, nutritional adequacy, and cardiometabolic risk.	Exposure markers are optional research measures; routine aging-clock screening is unsupported.
Primary care and metabolic services	Integrate dietary support with obesity, diabetes, cardiovascular care, and indicated fibrosis case finding.	Completion of risk assessment and referral, retention, metabolic control, and liver outcomes.	Use a prognostic or response biomarker only when its added information resolves a defined decision and has appropriate validation.
Specialist care and functional vulnerability	Adapt nutrition to fibrosis stage, cirrhosis, frailty, comorbidity, and medication effects.	Adequate intake, strength, mobility, quality of life, and clinically relevant liver outcomes.	Molecular response biomarkers remain subordinate to clinical benefit and functional safety; they are not surrogate endpoints.
Implementation and biomarker research	Compare equally supported clinical care with a validated biomarker-guided strategy.	Clinical net benefit, adherence, costs, assay feasibility, subgroup calibration, and equitable uptake.	Qualify exposure, response, prediction, and surrogate roles separately before adoption.

The framework synthesizes clinical guidance and the evidence discussed in this review [4,5,97]. Service and policy measures are proposed evaluation priorities, not validated effects of a specific delivery model. Routine clinical care does not require an aging-clock measurement.

**Table 8:** A qualification roadmap for aging measures in MASLD nutrition studies.

Qualification Step	Required Evidence	Appropriate Comparison or Design	Use Supported
Analytical validity	Observed change exceeds assay and within-person variability under standardized collection, processing, and model version.	Replicate samples, blinded assays, cross-platform comparison where relevant.	Reproducible measurement.
Biological validity	The signal reflects a defined aging-related process, with acute exposure, cell composition, injury, and repair distinguished.	Tissue-linked studies, repeated sampling, orthogonal biological measures, mechanistic experiments.	Interpretation of the construct.
Prognostic validity	The baseline measure predicts future clinically relevant outcomes beyond standard assessment.	Prospective cohorts; external calibration; comparison with fibrosis, metabolic, and functional measures.	Risk estimation for the validated population.
Longitudinal responsiveness	Nutrition produces reproducible change that persists beyond transient metabolic adaptation.	Randomized diet trial with a prespecified endpoint, matched sampling, and adequate follow-up.	Response monitoring in research.
Predictive utility	The baseline measure identifies different relative benefits from alternative diets.	Prespecified treatment-by-biomarker interaction; independent validation of the resulting rule.	Selection among tested strategies.
Surrogate validation	Treatment-induced changes reliably predict later clinical benefit across interventions.	Multiple suitable trials with clinical endpoints; temporal and causal evaluation beyond within-trial correlation.	Substitution for a clinical endpoint only within a validated context.
Clinical utility	Adding the measure improves outcomes compared with equally intensive standard care.	Randomized comparison of biomarker-guided and clinically guided nutrition, including harms and adherence.	Routine use if net benefit is demonstrated.
Cost-effectiveness and equity	Additional health benefits justify testing costs, with acceptable access and performance across relevant groups.	Economic evaluation alongside implementation; subgroup performance, uptake, and access assessment.	Decisions about service adoption.

This is a proposed qualification framework, not a validated clinical algorithm. Evidence of prognostic value, responsiveness, predictive utility, and surrogate validity supports different uses; success at one stage does not imply success at the others. Parallel work on several stages is appropriate, but the intended clinical claim determines the evidence required.

## Data Availability

No new data were generated for this narrative review. The evidence discussed is available in the cited publications, and the search strategies and selection audit are included in Appendices Appendix A and Appendix B. Further information regarding this review may be obtained from the corresponding authors upon reasonable request.

## Abbreviations

The following abbreviations are used in this manuscript:

MASLD	Metabolic dysfunction-associated steatotic liver disease
MASH	Metabolic dysfunction-associated steatohepatitis
NAFLD	Nonalcoholic fatty liver disease
NASH	Nonalcoholic steatohepatitis
DNAm	DNA methylation
KDM	Klemera–Doubal method
MRI-PDFF	Magnetic resonance imaging proton density fat fraction
CAP	Controlled attenuation parameter
FIB-4	Fibrosis-4 index
ELF	Enhanced Liver Fibrosis test
DNL	De novo lipogenesis
RCT	Randomized controlled trial
SASP	Senescence-associated secretory phenotype

## Appendix A. Search Strategy and Database Queries

This appendix records the search strategy used for this critical narrative review. It is intended to improve reproducibility and does not imply systematic-review completeness or pooled inference. Searches were conducted from 1 June to 15 September 2026 in PubMed/MEDLINE, Web of Science Core Collection, Scopus, and Google Scholar. Citation chasing of pivotal guidance and primary studies continued through 30 September 2026.

The main concept blocks were combined as follows: (MASLD OR NAFLD OR NASH OR “metabolic dysfunction-associated steatotic liver disease” OR “metabolic dysfunction-associated fatty liver disease”) AND (nutrition OR diet* OR dietary OR “weight loss” OR fiber OR Mediterranean OR “time-restricted” OR ketogenic) AND (metabolom* OR lipidom* OR proteom* OR epigen* OR “biological aging” OR senescence OR “aging clock” OR DunedinPACE OR fibrosis OR “liver fat”). Historical NAFLD/NASH terms were used to identify the original disease definition of earlier studies; current recommendations were framed for MASLD.

**Table A1:** Database-specific search strings used in the narrative review.

Database	Principal Search String	Date
PubMed/MEDLINE	(MASLD OR “metabolic dysfunction-associated steatotic liver disease” OR NAFLD OR NASH OR “metabolic dysfunction-associated fatty liver disease”) AND (nutrition OR diet* OR dietary OR “weight loss” OR fiber OR Mediterranean OR “time-restricted” OR ketogenic) AND (metabolom* OR lipidom* OR proteom* OR epigen* OR “biological aging” OR senescence OR “aging clock” OR DunedinPACE OR fibrosis OR “liver fat”)	June–September 2026
Web of Science	TS=((MASLD OR NAFLD OR NASH OR “metabolic dysfunction-associated steatotic liver disease”) AND (nutrition OR diet* OR “weight loss” OR Mediterranean OR fiber OR meal-timing) AND (metabolom* OR lipidom* OR proteom* OR epigen* OR aging OR senescence OR fibrosis))	June–September 2026
Scopus	TITLE-ABS-KEY((MASLD OR NAFLD OR NASH OR “metabolic dysfunction-associated steatotic liver disease”) AND (nutrition OR diet* OR “weight loss” OR Mediterranean OR fiber OR metabolom* OR epigen* OR “biological aging” OR fibrosis))	June–September 2026
Google Scholar	Exact-phrase and paired-keyword searches: “MASLD” + nutrition; “MASLD” + metabolomics; “NAFLD” + aging clock; “liver” + epigenetic age + diet; resistant starch + liver fat; Mediterranean + MASLD; DunedinPACE + liver; followed by cited-by and related-article tracing.	June–September 2026

## Appendix B. Eligibility, Selection, and Evidence Audit

Reports were eligible when they informed at least one of the following: a human MASLD/NAFLD–aging association; a nutritional intervention with hepatic or aging outcomes; validation or evaluation of an aging or metabolomic measure; a mechanistic pathway relevant to hepatic injury; or a clinical/public-health decision concerning fibrosis, metabolic risk, nutritional adequacy, or functional reserve. Animal and cell studies were retained only for mechanism and were not treated as clinical efficacy evidence. Guidance and consensus documents were retained for current terminology and care pathways. Reports were excluded when they lacked a relevant exposure, outcome, measurement method, or clinical implication; were duplicate records; or could not be interpreted from the available report.

One author screened titles and abstracts and reviewed full texts. A second author independently checked all included reports and all uncertain exclusions. Disagreements about eligibility, original disease definition, tissue source, biomarker role, or risk of overinterpretation were resolved by discussion with the senior author. Data were extracted into a structured worksheet covering population, original disease definition, design, exposure, sample/tissue source, analytical platform, outcome, replication, and clinical directness. Companion reports from the same trial were linked to the parent trial and were not counted as independent randomized populations.

The counts are audit estimates because databases overlap and this is a narrative review; domain totals are therefore not additive. The final synthesis emphasized design quality, explicit tissue source, outcome definition, replication, and translational relevance, and did not calculate a pooled treatment effect. Exposure markers document intake or adherence; association biomarkers relate a measurement to disease or pathology; prognostic biomarkers estimate future risk; response biomarkers change after intervention; and candidate surrogate endpoints require evidence that treatment-induced change predicts later clinical benefit across appropriate interventions. A result in one role was not interpreted as evidence for another role. Historical NAFLD/NASH labels were retained only for original study populations, while current recommendations were interpreted against the contemporary MASLD definition and graded as direct, indirect, or uncertain transportability.

**Table A2:** Approximate records screened and retained by evidence domain.

Evidence Domain	Records Screened	Reports Retained	Typical Retained Designs
MASLD/NAFLD and biological-aging associations	≈420	≈34	Cohorts, biopsy studies, prognostic models, case–control studies
Nutrition and aging measures outside MASLD	≈310	≈27	Randomized diet, calorie-restriction, omega-3, exercise, and observational aging studies
Nutritional interventions and hepatic outcomes	≈510	≈46	Randomized trials, prospective weight-loss cohorts, controlled feeding studies
Metabolomics, lipidomics, and molecular response	≈390	≈39	Controlled feeding, metabolomic profiling, mechanistic human studies
Precision nutrition, implementation, and guidance	≈260	≈31	Guidelines, care models, treatment-matching studies, public-health analyses
